# Genome-Scale Metabolic Model of *Xanthomonas phaseoli* pv. *manihotis*: An Approach to Elucidate Pathogenicity at the Metabolic Level

**DOI:** 10.3389/fgene.2020.00837

**Published:** 2020-08-11

**Authors:** David Botero, Jonathan Monk, María Juliana Rodríguez Cubillos, Andrés Rodríguez Cubillos, Mariana Restrepo, Vivian Bernal-Galeano, Alejandro Reyes, Andrés González Barrios, Bernhard Ø. Palsson, Silvia Restrepo, Adriana Bernal

**Affiliations:** ^1^Laboratory of Mycology and Plant Pathology (LAMFU), Department of Chemical and Food Engineering, Universidad de Los Andes, Bogotá, Colombia; ^2^Grupo de Diseño de Productos y Procesos (GDPP), Department of Chemical and Food Engineering, Universidad de Los Andes, Bogotá, Colombia; ^3^Max Planck Tandem Group in Computational Biology, Universidad de Los Andes, Bogotá, Colombia; ^4^Grupo de Biología Computacional y Ecología Microbiana, Department of Biological Sciences, Universidad de Los Andes, Bogotá, Colombia; ^5^Systems Biology Research Group, Department of Bioengineering, University of California, San Diego, San Diego, CA, United States; ^6^Department of Molecular Plant Physiology, Potsdam University, Potsdam, Germany; ^7^Laboratory of Molecular Interactions of Agricultural Microbes, LIMMA, Department of Biological Sciences, Universidad de Los Andes, Bogotá, Colombia

**Keywords:** *Xanthomonas*, *Xpm*, cassava bacterial blight, genome-scale metabolic model, quorum sensing

## Abstract

*Xanthomonas phaseoli* pv. *manihotis* (*Xpm*) is the causal agent of cassava bacterial blight, the most important bacterial disease in this crop. There is a paucity of knowledge about the metabolism of *Xanthomonas* and its relevance in the pathogenic process, with the exception of the elucidation of the xanthan biosynthesis route. Here we report the reconstruction of the genome-scale model of *Xpm* metabolism and the insights it provides into plant–pathogen interactions. The model, iXpm1556, displayed 1,556 reactions, 1,527 compounds, and 890 genes. Metabolic maps of central amino acid and carbohydrate metabolism, as well as xanthan biosynthesis of *Xpm*, were reconstructed using Escher (https://escher.github.io/) to guide the curation process and for further analyses. The model was constrained using the RNA-seq data of a mutant of *Xpm* for quorum sensing (QS), and these data were used to construct context-specific models (CSMs) of the metabolism of the two strains (wild type and QS mutant). The CSMs and flux balance analysis were used to get insights into pathogenicity, xanthan biosynthesis, and QS mechanisms. Between the CSMs, 653 reactions were shared; unique reactions belong to purine, pyrimidine, and amino acid metabolism. Alternative objective functions were used to demonstrate a trade-off between xanthan biosynthesis and growth and the re-allocation of resources in the process of biosynthesis. Important features altered by QS included carbohydrate metabolism, NAD(P)^+^ balance, and fatty acid elongation. In this work, we modeled the xanthan biosynthesis and the QS process and their impact on the metabolism of the bacterium. This model will be useful for researchers studying host–pathogen interactions and will provide insights into the mechanisms of infection used by this and other *Xanthomonas* species.

## Introduction

Cassava (*Manihot esculenta* Crantz) is one of the most important crops in Africa, Asia, and around the world due to its drought tolerance, its ability to grow on acidic soils and in low nutrient conditions, and its high tolerance against several pests and diseases (Lopez et al., 2016). Africa, Asia, and South America contribute 53.5, 30.2, and 15.6% of the world’s cassava production (Lopez et al., 2016). Additionally, cassava serves as a food source for animals and humans, and it is used for various industrial applications including starch for textiles, medicine, and alcohol production (FAO, 2000).

Cassava bacterial blight (CBB), caused by *Xanthomonas phaseoli* pv. *manihotis* (*Xpm*), previously known as *Xanthomonas axonopodis* pv. *manihotis* (Constantin et al., 2016), is the most important bacterial disease of cassava, causing yield losses ranging from 12 to 100% (Lozano, 1986). The main symptoms of CBB are leaf spot, drying, shriveling, and stem die-back (Lozano, 1975, 1986). Several molecular processes have been linked with pathogenicity and survival in the host plant. Among them, quorum sensing (QS), a process conserved in the genus *Xanthomonas*, regulates several virulence pathways, such as the production of extracellular enzymes, exopolysaccharide production, and flagellar synthesis, as well as resistance to toxins and oxidative stress (Dow et al., 2000; He et al., 2006; Guo et al., 2012). The diffusible signal factor (DSF)-mediated pathway has been widely studied in the genus *Xanthomonas*, chiefly in *Xanthomonas campestris* and *Xanthomonas citri* (Guo et al., 2012). In this pathway, RpfF, functioning as a putative enoyl-CoA hydratase, catalyzes the production of DSF, while the two-component system RpfC–RpfG is involved in the sensing and the transduction of the DSF signal, at least in *X. campestris* pv. *campestris* (*Xcc*) and *X. citri* (*Xci*) (Andrade et al., 2006; He et al., 2006; Huang et al., 2013). These three genes have a high degree of conservation among *Xanthomonas* species and affect multidrug and hydrogen peroxide resistance, iron uptake, flagellar genes, and exopolysaccharide biosynthesis (EPS) (He et al., 2007; Guo et al., 2012). An additional gene, *rpfH*, is also found in some species of *Xanthomonas*, including *Xpm* (Arrieta-Ortiz et al., 2013), although its role still remains elusive (Barber et al., 1997; Dow et al., 2000). Additional receptors of the DSF signal have been reported in *Xcc* (An et al., 2014), but what they control is not well known. Besides DSF, another signaling molecule synthesized by *pigB*, termed DF, has also been described in *Xanthomonas* and linked to the production of EPS and the ultraviolet-protecting pigment known as xanthomonadin (Poplawsky and Chun, 1997, 1998a,b). *Xanthomonas campestris* mutants for *rpfC*, *rpfG*, and *rpfF* also show a reduction in virulence when compared to wild-type phenotypes (Guo et al., 2012). Nevertheless, most of the information regarding the regulation of virulence genes related with quorum sensing in *Xpm* come from extrapolation of studies performed on related species. While they might enable predictions, it is crucial to validate and identify the virulence determinants of this bacterium. Moreover, QS influences the biosynthesis of xanthan, the major exopolysaccharide produced by *Xanthomonas* (Dow et al., 2000; Vojnov et al., 2001; Torres et al., 2007). This major virulence factor is produced at higher population densities, which generally correlate with the invasion and successful multiplication within the host, and it is required for biofilm formation (Li and Wang, 2011). However, the effect of QS on other metabolic routes has not been studied. This is important since the nutritional aspect, although understudied in plant pathogens, is pivotal in the interaction of a pathogen with its host. The large amount of data produced by next-generation omic technologies is an opportunity to advance in the knowledge of this pathosystem. In *Xanthomonas*, several studies have been performed in the fields of transcriptomics, metabolomics, population genomics, and phylogenomics (Gordon et al., 2015; Kogenaru et al., 2012; Liu et al., 2013; Rodriguez-R et al., 2012; Schatschneider et al., 2011; Schmidtke et al., 2012). For example, the metabolic pathway for the production of xanthan (a polysaccharide secreted by bacteria in the genus *Xanthomonas*, which has been involved in pathogenicity and has been widely used in the industry as a food additive) has been elucidated *in silico* and confirmed through experimental verification (Schatschneider et al., 2013). More specifically in *Xpm*, population genetics, phylogenetic relationships, genome analyses, and forward and reverse genetics have been performed with the aim of uncovering the population structure of this plant pathogen and to search for pathogenicity factors (Arrieta-Ortiz et al., 2013; Bart et al., 2012; Castiblanco et al., 2013; Cohn et al., 2014; Cohn et al., 2016; Medina et al., 2018; Restrepo et al., 1999; Trujillo et al., 2014a, b). However, until now, none of these studies have used systems biology approaches to elucidate the pathogenicity mechanisms in *Xpm*.

Systems biology has enabled the investigation of organisms as a whole. Within this field, metabolic processes in human bacterial pathogens have been studied using the Constraint-Based Reconstruction and Analysis (COBRA) approach (Bartell et al., 2014; Fong et al., 2013; Liao et al., 2011; Mithani et al., 2011; Steinway et al., 2015; Thiele et al., 2011). Among plant bacterial pathogens, this approach has been used to study the metabolic processes involved in the pathogenicity of *Xcc* (Schatschneider et al., 2013), *Pseudomonas syringae* pv. *tomato* (Ward et al., 2010), *Pectobacterium carotovorum* (Wang et al., 2014), and *Ralstonia solanacearum* (Peyraud et al., 2016).

In this study, the metabolic model of *Xpm* at the genome scale was developed. The model was integrated with RNA-seq data from *rpfCGH* mutants of *Xpm*. Differential gene expression of mutant strains was used to construct context-specific metabolic models (CSMs) using the COBRA approach. This metabolic model is an approach to understand the mechanisms of metabolic pathways directly or indirectly related to the quorum sensing and xanthan biosynthesis processes of *Xpm*. Specifically, we aimed at determining the metabolic map of carbohydrate utilization pathways in *Xpm* as a first step to understand its nutritional relationship with the host plant. In addition, we hypothesized that the xanthan biosynthesis route has an energetic cost that the bacterium must pay in order to survive in the environment and cause disease.

## Materials and Methods

### Metabolic Model of *Xanthomonas phaseoli* pv. *manihotis*

The most complete and best annotated genome of *Xpm* (Arrieta-Ortiz et al., 2013), from strain CIO151^1^, was used to build a metabolic model of the bacterium at the genome scale. This metabolic reconstruction was performed by two complementary approaches: one based on different metabolic databases and a second one using the modelSEED server (Devoid et al., 2013), followed by manual curation.

First, enzyme-coding genes and their Enzyme Commission (EC) numbers were extracted from the annotation file of *Xpm* (657 EC from 4,340 proteins). These were used to retrieve candidate metabolic reactions from three databases: MetaNetx (Ganter et al., 2013), KEGG (Kanehisa and Goto, 2000), and BiGG (Schellenberger et al., 2010). The reactions obtained were subsequently used to construct draft metabolic models based on each database.

To supplement these draft models, the genome was uploaded to the modelSEED server and an automatic draft metabolic model was reconstructed. This model was converted to BiGG standard format using MetaNetX. Next, we performed comparisons between the reactions of the different reconstructions (using MetaNetX as cross-reference) in order to determine the number of reactions in common between the reconstructions and the level of agreement with the modelSEED reconstruction. Network gaps were identified and filled using the GapFind and GapFill algorithms (Satish Kumar et al., 2007). Finally, the combined model was manually curated *via* literature review and Escher, a visualization tool for metabolic pathways (King et al., 2015). Forty-one reactions were added, including 16 in the xanthan biosynthesis and 20 in the amino acid metabolism routes. Five reactions were deleted; the reversibility of four reactions and the stoichiometry of two reactions were changed. The reviewed literature can be found in the Supplementary Material (Supplementary Data Sheet S1) and includes 62 entries related with 38 reviewed articles as well as specifications and notes of the items curated in the metabolic network. The number of proteins associated with metabolic functions was higher for modelSEED (890) than that obtained solely by enzyme codes (386).

Several reactions important for carbohydrate source assimilation, central metabolism, xanthan biosynthesis, and amino acid metabolism were absent in the metabolic model and were identified during the literature review procedure and using the existing annotation. Additionally, amino acid pathways were manually gapfilled using KEGG, MetaCyc, and BiGG databases. We corrected the directionality of reactions. Special interest was put in exchange reactions to define minimal medium and carbon sources. In order to find the sequences of the missing genes in the *Xpm* genome, a BLAST search (Camacho et al., 2009) looking for sequences in the *Xpm* genome with a high similarity to the genes related with the missing reaction (e-value smaller than 1E-6) reported in BiGG database was performed. In the case of the xanthan biosynthesis pathway, the backbone of the pathway was constructed based on the existing map reported for *Xcc* available in MetaCyc and KEGG and previously modeled (Schatschneider et al., 2013, 2011; Vorhölter et al., 2008).

Flux balance analysis (Orth et al., 2010) was used to simulate bacterial growth under different conditions. Three hundred thirty-one reactions involved in 124 loops (unfeasible thermodynamic cycles) were identified using the null space of the stoichiometric matrix and the CycleFreeFlux (Desouki et al., 2015) algorithm implemented in COBRApy (Ebrahim et al., 2013) in order to improve the metabolic model. The model was tested with and without loops using CycleFreeFlux without any change in the growth rate. Additionally, a restriction in the directionality of the reactions identified through the null space was used to test changes in the response of the model, but there were no changes in growth rate nor a reduction of the number of loops. Therefore, we only report the number of loops. Data related with the identified loops by CycleFreeFlux are reported in Supplementary Table S1; flux variability analysis was performed in the model produced by this algorithm and compared with the original model in order to identify the loops. Data related with identified loops by null space are shown in Supplementary Table S2. The metabolic model with all the exchange reactions open was used; those reactions with values different to zero in every column belong to the same cycle.

### Merging Annotation

Because the annotation performed by modelSEED server provides a gene ID and the transcript abundances were calculated using the genome annotation performed by Arrieta-Ortiz et al. (2013), the two annotations were merged using the BLAST Reciprocal Best Hit tool (Supplementary Table S3). This tool was used to search for the best hits between the genes of the two annotation files (modelSEED and Arrieta et al.) (Camacho et al., 2009; Cock et al., 2015). The parameters used by default to filter hits out were minimum percentage identity of 70%, minimum coverage of 50%, and minimum high-scoring segment pair coverage of 50%. Then, the best hit for each gene was used for merging the annotations.

### Objective Function

Simulations of growth were performed using the maximization of the rate of the biomass reaction as objective function; the stoichiometric coefficients for this reaction can be found in Supplementary Table S4. Additionally, an alternative objective function of biomass and xanthan production was used based on the previous modeling work of *Xcc* (Schatschneider et al., 2013; Vorhölter et al., 2008). The exopolysaccharide xanthan was included into the alternative objective function for two reasons: first, in order to perform simulations of the response of the model to the optimization of this pathogenicity and survival factor and, second, we aimed at testing the computational feasibility of the inclusion in the model of a pathogenicity factor, as was recently done for another vascular plant pathogen (Peyraud et al., 2016).

The alternative objective function (Biomass2) of biomass and xanthan corresponds to the addition of the metabolites xanthan and biomass:

B⁢i⁢o⁢m⁢a⁢s⁢s⁢2=0.8⁢x⁢a⁢n⁢t⁢h⁢a⁢n+0.2⁢b⁢i⁢o⁢m⁢a⁢s⁢s

We tested both objective functions: biomass and biomass with xanthan. This proportion of xanthan and biomass (80–20%) was experimentally determined in *Xcc* (Vorhölter et al., 2008). Finally, computational simulations of the response of the growth rate to the change of the proportion of xanthan to biomass were performed in order to determine if there is a trade-off between xanthan and growth. The xanthan proportion was changed in steps of 0.5% from 0 to 100%, and the resulting growth rate was registered.

### *Xpm* Strains for RNA-Seq Experiments

Two strains derived from *Xpm* strain CIO151 were used in this study: (1) CIO151 strain transformed with the empty vector pBBR1-MCS5 (*Xpm* CIO151 EV) and (2) a double recombinant mutant previously generated (M. Restrepo et al., 2012) lacking *rpfC*, *rpfG*, and *rpfH* and transformed with the empty vector pBBR1-MCS5 (*Xpm* CIO151 Δ*rpfCGH*-EV).

In order to determine the time-point to perform the RNA extraction, a common exponential growth phase among strains was established by means of a growth curve. The growth assay was performed using NYG complex medium (3 g/L yeast extract, 5 g/L peptone, and 30 g/L glycerol, pH 7.0) for liquid cultures and NYGA (NYG containing 15 g agar l^–1^) for plating serial dilutions of the liquid cultures. The cells were plated at 4-h interval time-points between 0 and 24 h. Because the wild-type and the mutant strains showed the same growth rate, we selected 18 h as the time for mid- to late-exponential phase for the RNA extraction.

### RNA Extraction and RNA-Seq Analysis

Total RNA was extracted using a modified hot phenol-chloroform manual extraction method (Jahn et al., 2008). Briefly, each strain was grown on solid medium (NYGA), and a single colony was subcultured in 30 ml of NYG medium and incubated in constant agitation for 18 h from an initial OD_600_ of 0.002; the cells were harvested at 18 h. The cells were then centrifuged, placed on ice, and mixed with hot acid phenol (pH 4.5) and a lysis buffer solution consisting of 4 M LiCl, SDS 2%, 0.5 M EDTA (pH 8), and 1 M Tris–HCl (pH 8). The cells were then vortexed, and a solution of chloroform/isoamyl alcohol (24:1) was added prior to a second vortexing and centrifugation step to separate the different phases. Two overnight precipitation steps were carried out: the first with 4 M lithium chloride and the second one with 3 M sodium acetate (pH 5.2). RNA quality was confirmed using a Bioanalyzer (Agilent 2100, Santa Clara, CA, United States). Two independent extraction processes were performed with different colonies for each strain to account for two biological replicates. Subsequent procedures were performed at the Beijing Genome Institute (Hong Kong): rRNA depletion was performed with the Ribo-Zero^TM^ rRNA Removal Kit from Epicentre (Madison, WI, United States), and RNA-Seq was performed through the Illumina HiSeq 2000 platform. The libraries generated were paired-end (100-bp read length) and strand specific (Parkhomchuk et al., 2009). Raw data have been deposited in NCBI under the project ID PRJNA598165 and SRA codes SRX7570863–SRX7570866.

An average of 35 million reads was obtained per sample. The sequencing quality of the reads was visualized using FASTQC, available at http://www.bioinformatics.babraham.ac.uk/projects/fastqc/(Van Verk et al., 2013). The reads were trimmed and filtered with FASTX-Toolkit^2^ (Supplementary Table S5). To ensure the removal of contaminating ribosomal RNA (rRNA), all reads aligning to indexed rRNA sequences of *Xpm* were discarded using the Bowtie2 aligner (Langmead and Salzberg, 2013). Approximately 18% of reads in each library were lost due to low quality during the filtering process, and a subsequent 11% was removed after *in silico* cleaning of rRNA, leaving an average of 27 million reads per sample (Supplementary Table S5). The clean reads were aligned against the *Xpm* CIO151 genome (Arrieta-Ortiz et al., 2013) using TopHat (Trapnell et al., 2009), and the transcripts were counted using Cufflinks (Trapnell et al., 2012). The normalized abundance, in fragments per kilobase of transcript per million mapped reads (FPKM), was used to identify differentially expressed genes with NOISeqBIO (Tarazona et al., 2011) and R Bioconductor package using a threshold of FDR < 0.05 (Tarazona et al., 2011). Subsequently, the differentially expressed genes (DEGs) were annotated according to Gene Ontology (GO), and overrepresented GO terms were distinguished using Blast2GO^®^.

Based on previous reports from other *Xanthomonas*, *rpfC* and *rpfG* along with 11 *gum* genes involved in extracellular polysaccharide production and biofilm formation were used as positive controls for the differential expression data from *Xpm* CIO151 ΔrpfCGH-EV (Guo et al., 2012; He et al., 2007; Slater et al., 2002) to compare with the DEGs obtained with NOISeqBIO.

### qRT-PCR Validation

qRT-PCR was used for the validation of a subset of differentially expressed genes among strains. SsoFast^TM^ EvaGreen^®^ Supermix (Bio-Rad) was used with the 7500 FAST (Applied Biosystems) thermal cycler according to the manufacturer’s instructions; melting curves were used in each run to detect and discard experiments with non-specific amplifications. *gyrB* was selected as a housekeeping gene for validation because it showed low variability compared with *rpoB*, *rpoD*, and *atpD* genes (data not shown). The Pfaffl method (Huber et al., 2015) was employed for relative quantification, with the use of standard curves for the determination of efficiency rates between target genes; only efficiencies between 90 and 110% were accepted. The RNA-Seq and the qRT-PCR results for the validated genes were then evaluated through a Pearson correlation to determine if both sets of data supported each other.

### Context-Specific Models of *Xpm* and Simulations

The iMAT algorithm (Zur et al., 2010), implemented in COBRA Toolbox v3.0 (Pfaffl, 2001), was used to generate context-specific models of *Xpm* CIO151 EV and *Xpm* CIO151 Δ*rpfCGH*-EV. iMAT can use the discretized values of the expression values to classify the reactions into highly, moderately, and lowly expressed reaction groups. The average of abundance per replicate of the transcripts for the genes of the two strains, normalized by FPKM calculation, was used for the discretization. Although the iMAT authors recommend using mean and half standard deviations of the transcript abundances to delimit the high and the low thresholds of the data and establish the set of the switch-on and switch-off genes for the reactions, it was determined that this method would give a negative low threshold in our case because the transcript abundances of *Xpm* for the two conditions do not have a normal distribution. Therefore, the first and the third quartiles of these abundances were calculated in MATLAB^®^ and used as low and high thresholds to define the genes with high, moderate, and low expression for each strain. Supplementary Table S6 summarizes the statistics of the expression data. Discretization was performed by assigning a value of −1, 0, and 1, respectively, to low, moderate, and high groups of genes. Then, the discretized values of the genes were mapped to the reactions using the gene reaction rules of the model (using the COBRA Toolbox). The iMAT algorithm was executed and the CSMs were generated for both *Xpm* CIO151 EV and *Xpm* CIO151 Δ*rpfCGH*-EV. Finally, flux balance analysis (FBA) for each CSM using both biomass and biomass and xanthan as objective functions was performed.

### Bacterial Growth for Wild Type and Mutant Comparison

In order to compare the growth rate of the two strains, testing of the *in vitro* growth of the mutant and the wild type in Phi ϕ liquid media for a period of 28 h at 28°C with shaking at 200 rpm was performed. The cell populations were determined by measuring their colony-forming units (CFU) in dilution plating (growth in LPGA solid media for 48 h at 28°C) at different points of the Phi ϕ liquid culture (10 g/L of peptone, 1 g/L of yeast extract, and 1 g/L of casamino acids). The composition of plating LPGA solid media was 5 g/L of yeast extract, 5 g/L of peptone, 5 g/L of glucose, and 15 g/L of agar. Rifampicin antibiotic (100 μg/ml) was added to the media.

### Hierarchical Clustering Heatmap

In order to group the flux of reactions for the different strains, a hierarchical clustering of CSMs for the strains using the two objective functions, biomass and biomass and xanthan production, was performed. First, the reactions with value of fluxes equal to zero for all the conditions were deleted. Then, normalization of flux values was computed using *Z*-score. The Ward method was used to calculate the linkage matrix based on Euclidean distance (Zur et al., 2010). Cluster maps of the two strains under the two objective functions were calculated. For coloring purposes, a dendrogram was constructed using a grouping color threshold of 25% of the branch length. The calculations were performed in Python using SciPy package.

### Pathway Visualization

Pathways for carbohydrate source assimilation, glycolysis, central metabolism, xanthan biosynthesis, and amino acid metabolism were constructed in Escher (Heirendt et al., 2018). The maps for these pathways can be downloaded from GitHub^3^. Transcriptomic abundances were mapped into Escher maps. A purple color in the maps corresponds to low abundance and a red color to high abundance. The median of the transcript abundances defined the middle point of coloring (between red and purple). Therefore, values below the median are colored purple and above the median red. When in the reaction rule two or more genes are strictly necessary (AND Boolean rule), the mean of the abundance was calculated. When in the reaction rule two or more genes code for the same reaction (OR Boolean rule), the sum of the abundances was calculated. The thickness of the lines is proportional to the abundance of the transcripts involved in the given reaction.

### Availability of Code and Data

All the code, maps, and metabolic models used have been uploaded in GitHub at https://github.com/davidoctaviobotero/Xpm_metabolic_model.

## Results

### Metabolic Model of *Xanthomonas phaseoli* pv. *manihotis* Is Similar to That of *Xcc* but Shows New Features Not Previously Reported for *Xpm*

**FIGURE 1:**
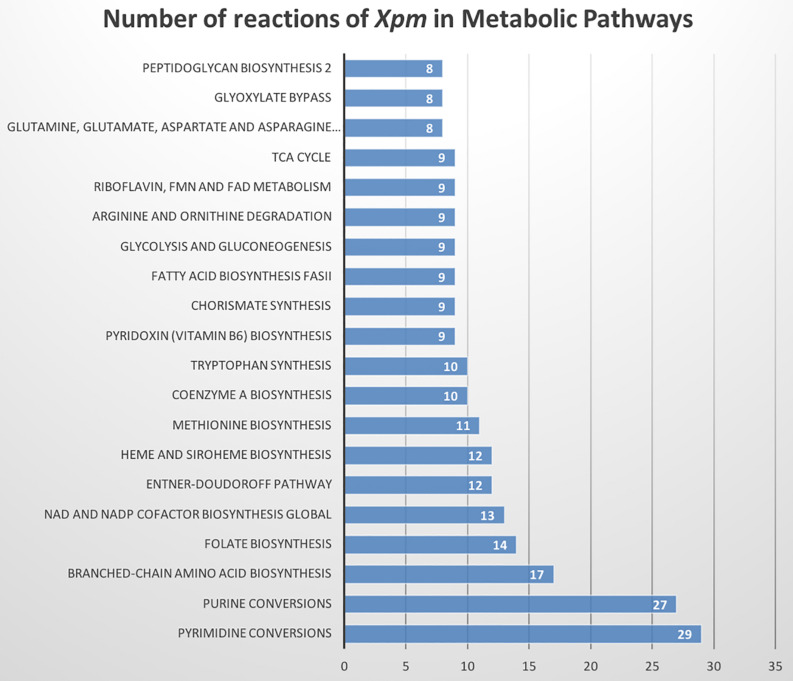
Metabolic pathways of *Xpm* CIO151. Bar graph of numbers of reactions in *Xpm* strain CIO151 assigned to each metabolic pathway. Only the top 20 most represented metabolic pathways are presented.

The metabolic model of *Xpm* CIO151, iXpm1556, had 1,556 reactions after curation, of which 155 were transport reactions and 90 were exchange reactions. The model included 1,527 metabolites, 890 genes associated with reactions, and 452 reactions (of the 1,556 total reactions) that could be assigned to 113 metabolic pathways of KEGG (Supplementary Table S7). Figure 1 shows the number of reactions assigned to each metabolic pathway for the 20 most represented. Pyrimidine conversion and purine conversion are the most highly represented pathways in *Xpm* CIO151, followed by amino acid and folate biosynthesis. Other pathways related with amino acid metabolism were also found. The amino acid metabolic map can be downloaded from GitHub^4^ (constructed in Escher).

**FIGURE 2:**
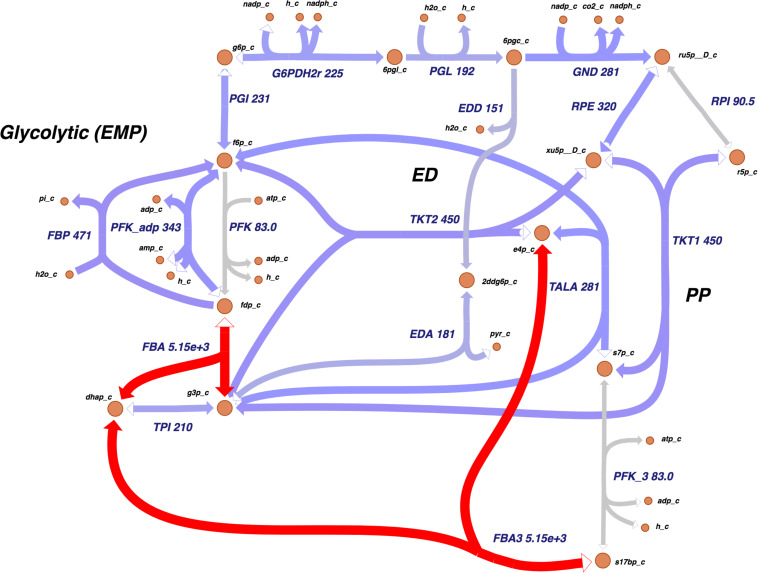
The carbohydrate utilization pathways of *Xpm* show a high activity at the transcriptional level. Transcriptomic data showed that *Xpm* has a high activity in the flux balance analysis (gene 1185660.4.peg.3367) and FBA3 (gene 1185660.4.peg.3367) reactions. The data of *Xpm* CIO151 EV are shown, but similar results were found for *Xpm* CIO151 Δ*rpfCGH-EV*. The levels of transcription are shown next to the name of the reaction. Transcriptomic abundances were mapped into Escher maps. The purple color in the maps corresponds to low abundance and the red color to high abundance. The median of the transcript abundances (FPKM) defined the middle point of coloring (between red and purple). Therefore, values below the median are colored purple and those above the median are colored red. When in the reaction rule two or more genes are strictly necessary (AND Boolean rule), the mean of the abundance was calculated. The thickness of the lines is proportional to the abundance of the transcripts involved in the given reaction. Entner–Doudoroff (ED), pentose-phosphate (PP), and Embden–Meyerhof–Parnas (EMP). Constructed in Escher (King et al., 2015).

The Entner–Doudoroff (ED) pathway, important in carbohydrate utilization metabolism and EPS production, also had a notable amount of reactions assigned. Two other main carbohydrate utilization pathways were found in *Xpm:* pentose-phosphate and Embden–Meyerhof–Parnas. Moreover, a carbohydrate source connected with the ED pathway, xylose, was found, which was previously reported as carbohydrate source in *Xcc* and as being involved in xanthan production (Ward, 1963). Figure 2 shows the map of carbohydrate utilization pathways.

**FIGURE 3:**
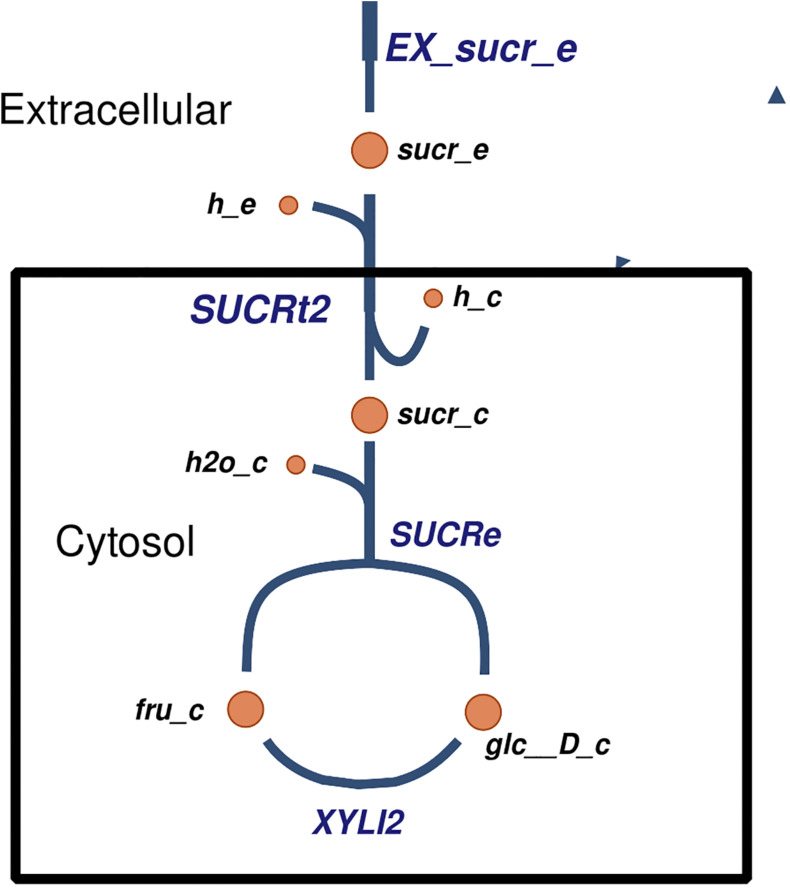
The proposed mechanism of transport of sucrose in *Xpm*. Sucrose is transported into the cell, where it is degraded into fructose and glucose. Constructed in Escher (King et al., 2015).

Several carbohydrate and nitrogen sources were predicted to be transported and metabolized by *Xpm*. For example, sucrose has been reported to be imported (King et al., 2015), but the exact mechanism of transport was not clear. In our model, we propose a mechanism of sucrose transport based on literature evidence and genome annotation. First, sucrose is imported into the cell *via* proton symport, and then it is hydrolyzed into glucose and fructose (Figure 3). Other predicted carbohydrate sources that are transported and metabolized in *Xpm* include fumarate (Zhang and Chen, 2010; Déjean et al., 2013), citrate, maltose, trehalose, arabinose, xylose (Van den Mooter et al., 1987), and D-mannose transported *via* the phosphoenolpyruvate (PEP)-dependent phosphotransferase system (PTS). Eleven nitrogen sources are predicted to be exchanged in the *Xpm* metabolic model. Three of them constituted inorganic forms and nine were organic (amino acids). L-arginine, L-lysine, and L-tryptophan (Zimaro et al., 2011) exchanging is essential for the growth of *Xpm* (from 20 essential metabolites determined *in silico* for the whole model). In the case of L-glutamine ABC transporter, evidence of glutamine transport at the genomic and the transcriptomic level was found in our model, however, there is no evidence in the literature for *Xpm*. BLAST of the glutamine transport ATP-binding protein of *Escherichia coli* against *Xpm* genome significantly hit several proteins previously annotated as ABC amino acid transporters. Other nitrogen sources transported in *Xanthomonas* are L-aspartate, L-glutamate, L-methionine, and L-proline (Ogunjobi et al., 2007; Zhang and Chen, 2010; Déjean et al., 2013) our model also predicted the transport of these nitrogen sources.

### Xanthan Biosynthesis Simulations in *Xpm* Show a Trade-Off Between Growth and Exopolysaccharide (Xanthan) Biosynthesis

**FIGURE 4:**
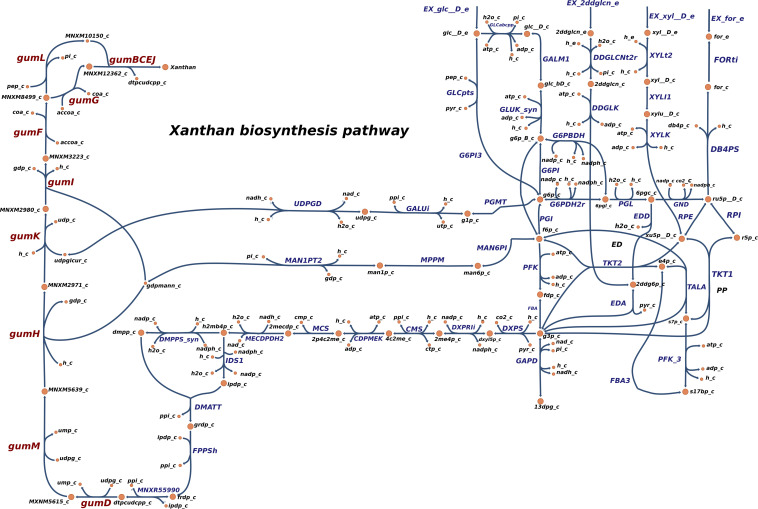
Reconstructed metabolic map of xanthan biosynthesis in *Xpm*. Reactions belonging to xanthan biosynthesis pathway are shown to the left side: gumD,M,H,K,I,F,G,L and gumBCEJ. The carbohydrate utilization pathways are connected with the xanthan biosynthesis reaction by three branches (middle part of the graph). PGMT, phosphoglucomutase; GALUi, UTP-glucose-1-phosphate uridylyltransferase; UDPGD, UDPglucose 6-dehydrogenase; MAN6PI, mannose-6-phosphate isomerase; MPPM, D-mannose 6-phosphate 1,6-phosphomutase; MAN1PT2, mannose-1-phosphate guanylyltransferase; DXPS, 1-deoxy-D-xylulose 5-phosphate synthase; DXPRIi, 1-deoxy-D-xylulose reductoisomerase; CMS, 2-C-methyl-D-erythritol 4-phosphate cytidylyltransferase; CDPMEK, 4-(cytidine 5′-diphospho)-2-C-methyl-D-erythritol kinase; MCS, 2-C-methyl-D-erythritol 2,4-cyclodiphosphate synthase; MECDPDH2, 2C-methyl-D-erythritol 2,4 cyclodiphosphate dehydratase; DMPPS, 1-hydroxy-2-methyl-2-(E)-butenyl 4-diphosphate reductase; IDS1, isopentenyl-diphosphate synthase; DMATT, dimethylallyltranstransferase; FPPSh, farnesyl pyrophosphate synthase; MNXR55990, undecaprenyl diphosphate synthase. Carbohydrate utilization pathways: Entner–Doudoroff (ED), pentose-phosphate (PP), and Embden–Meyerhof–Parnas (EMP). Constructed in Escher (King et al., 2015).

**FIGURE 5:**
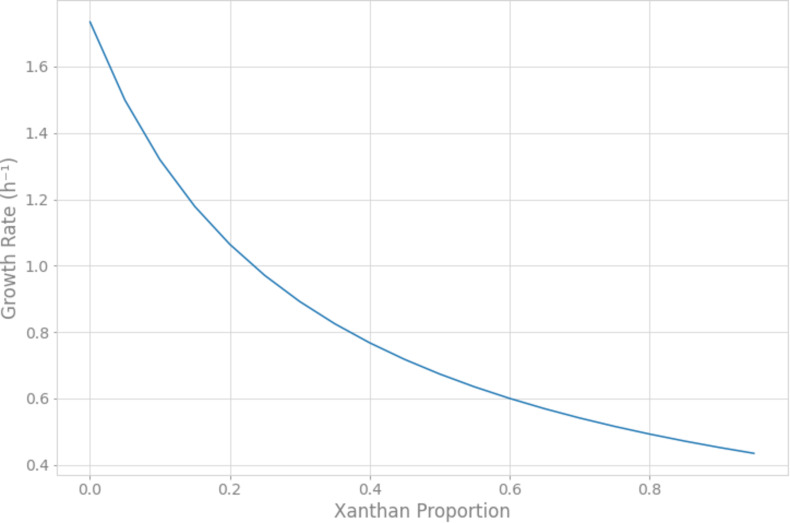
Trade-off between xanthan production and growth rate. The simulation of the response of the growth rate to the change in the xanthan proportion to biomass was performed through flux balance analysis. The xanthan proportion corresponds to the stoichiometric coefficient defined in the alternative objective function for xanthan metabolite. The growth rate decreases when xanthan production increases. The growth rate does not go down to zero because xanthan is part of the biomass function.

In order to simulate the physiological phenotype of growth for *Xpm* in the presence of a carbohydrate source, FBA was performed, using biomass as an objective function. The growth rate for *Xpm* in minimal medium with aerobic conditions and glucose as carbohydrate source was 1.73 h^–1^. In addition to biomass, xanthan production was modeled in *Xpm.* First, the xanthan biosynthesis pathway was reconstructed and manually curated (Figure 4, map provided in https://github.com/davidoctaviobotero/Xpm_metabolic_model/blob/master/Maps/Xpm-Xanthan_biosynthesis_map.json). The metabolic reconstruction of this pathway shows that all genes present in the *gum* cluster of *Xpm* are required for the last steps of xanthan biosynthesis. Furthermore, a strong connection between the reactions of the xanthan pathway and the carbohydrate utilization pathways was found through the branched reactions catalyzed by phosphoglucomutase, mannose-6-phosphate isomerase, and 1-deoxy-D-xylulose 5-phosphate synthase. Finally, a modified objective function of biomass with xanthan production was used to simulate the trade-off between biomass and xanthan biosynthesis. Figure 5 shows the change in the growth rate as response to the change in the proportion of xanthan to biomass. This simulation demonstrates the trade-off between growth and xanthan production; the growth rate decreases when xanthan production increases. The value for the growth when the *Xcc* experimentally measured proportions of biomass (80%) and xanthan (20%) are used together as the objective function was 0.493 h^–1^. This value is in the same order of magnitude as the experimental value for *Xpm* (0.43 h^–1^ maximal growth rate for a time period from 0 to 4 h in NYG medium, Supplementary Table S8) or for *E. coli* in glucose (0.9 h^–1^) (Swings and Civerolo, 1993) and in acetate (0.32 h^–1^) (Rojas et al., 2013; Swings and Civerolo, 1993). The difference between the two values of growth rate, 1.73 h^–1^ without and 0.493 h^–1^ with xanthan, highlights the importance of modeling the biosynthesis of pathogenicity and survival factors in bacterial plant pathogens.

### The Disruption of QS Results in Gene Expression Changes in *Xpm*

In order to test the model in the context of bacterial QS, we first used RNA-Seq to determine alterations in gene expression when the sensing of the QS signal is disrupted. Deletion of *rpfCGH* caused an alteration in the expression of 1,553 genes, out of which 99% corresponded to upregulated genes by QS pathway (downregulated in the *Xpm* CIO151 Δ*rpfCGH*-EV mutant; Supplementary Tables S9, S10), a global expression pattern that coincides with previous results published by Guo et al. (2012) for single mutants of *rpfC* and *rpfG* in *Xanthomonas citri* subsp. *citri*. Eleven *gum* genes involved in EPS production, together with *rpfC*, *rpfG*, and *rpfH*, were downregulated in the mutant strain *Xpm* CIO151 ΔrpfCGH-EV as expected, agreeing with previous results found for other *Xanthomonas* (Slater et al., 2002; He et al., 2007; Guo et al., 2012).

In order to elucidate the biological and the molecular processes affected by RpfCGH, we conducted a Fisher enrichment analysis for the DEGs using Blast2GO. Due to the low number of downregulated DEGs, no enrichment analysis was done for these genes. The altered molecular functions for *Xpm* CIO151 Δ*rpfCGH*-EV included previously reported roles influenced by DSF-QS, including bacterial-type flagellar motility, chemotaxis, signal transducer activity, and oxidoreductase activity. Motor, flagellar, and chemotaxis activities, along with signaling transduction, were among the most affected biological processes (Supplementary Figures S1, S2). Besides the *gum* genes involved in EPS biosynthesis, genes involved in the Type 3 Secretion System (T3SS), mainly *hrcS* and *hpa3*, were also downregulated in *Xpm* CIO151 Δ*rpfCGH*-EV. These results suggest that DSF-QS could be a positive regulator of the T3SS, as observed for HrpG, a master regulator of this system.

Out of the many RpfCGH-upregulated genes, we detected many interesting targets with hypothetical roles in phosphorylation sensor and transduction pathways for further investigation, some of which share a c-di-GMP phosphodiesterase activity (HD-GYP domain), just as RpfG. This could enable them to start different signaling cascades triggered by second messengers like c-di-GMP.

Genes previously not associated with pathogenesis in *Xanthomonas* and with roles that might prove useful during the infectious process were selected for validation using qRT-PCR. Prior to the validation process, four previously reported housekeeping genes were tested between our samples, and *gyrB* was selected as the preferred endogenous control.

From the comparison between *Xpm* CIO151 Δ*rpfCGH*-EV and *Xpm* CIO151-EV, a subset of putative sensor proteins (PhoB and a methyl-accepting chemotaxis protein) and a subset of regulatory proteins (a putative signal protein with an HD-GYP domain, a putative regulatory protein of a two-component system and an RNA polymerase sigma factor for the flagellar operon FliA) were selected for validation, using *gyrB* as the endogenous control (Supplementary Table S11). The qRT-PCR confirmed that all the selected genes were downregulated in the mutant strain (Supplementary Figure S3). Additionally, the Pearson coefficient showed a significant correlation at the 0.05 level between the RNA-Seq and qRT-PCR data, meaning that both sets of results supported one another (Supplementary Figure S4).

### Metabolic Functions of *Xpm* Are Altered by the Sensing Process

The metabolic model of *Xpm* was used in the context of bacterial quorum sensing. First, the normalized expression data of RNA-seq experiments for two *Xpm* strains were mapped to Escher metabolic pathways. Second, CSMs constructed with the expression levels of the two strains were analyzed. Finally, FBA and hierarchical clustering of flux values were depicted.

A total of 801 metabolic genes (80% of the genes) were associated with reactions with evidence at the transcript level. This validated 1,268 (81%) of the reactions constructed in our model at the transcription level. A total of 219 metabolic genes covered in the model were differentially expressed: 211 upregulated and eight downregulated by the Rpf-dependent QS pathway (Supplementary Table S12). Important subsystems at the metabolic level which were differentially expressed included central carbohydrate metabolism, amino acid, fatty acid, carbohydrate utilization, stress, and EPS metabolism. Some of the relevant differentially expressed genes altered by QS in *Xpm* include formate production reaction and D-mannose transport *via* PEP:Pyr PTS, transport of fumarate and citrate, and metabolism of maltose and trehalose which were repressed in the *rpfCGH* mutant.

NAD metabolism was of special interest because of the high percentage of reactions being differentially expressed (a total of 29 reactions out of 259 involved in the metabolism of NAD; Supplementary Table S13). Among them, NAD(P)^+^ transhydrogenase was inactive in the mutant of QS. The differentially expressed reactions mainly belong to pathways related with the biosynthesis of antibiotics and amino acids, carbon and secondary metabolism, and nicotinate and nicotinamide metabolism (Supplementary Table S14). Other pathways included purine metabolism, glycolysis and gluconeogenesis, propanoate, and glutathione.

**FIGURE 6:**
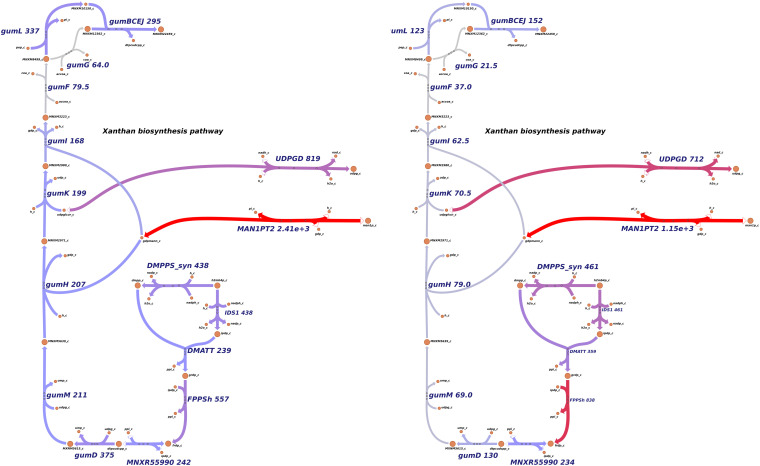
Differential expression of genes involved in xanthan production. Levels of transcript abundance were mapped to the metabolic pathway of xanthan. (A) *Xpm* CIO151 EV. (B) *Xpm* CIO151 Δ*rpfCGH-EV*. Xanthan production genes were downregulated in *Xpm* CIO151 Δ*rpfCGH-EV* when compared with *Xpm* CIO151 EV and analyzed in NOISeqBIO (King et al., 2015). The transcript abundance of *Xpm* CIO151 Δ*rpfCGH-EV* is half of that of the *Xpm* CIO151 EV. Seven gum genes (gumM, gumK, gumI, gumH, gumG, gumF, and gumD) were upregulated by the quorum sensing pathway. Transcriptomic abundances were mapped into Escher maps. The purple color in the maps corresponds to low abundance and the red color to high abundance. The median of the transcript abundances (FPKM) defined the middle point of coloring (gray). Therefore, values below the median are colored purple and above the median red. When in the reaction rule two or more genes are strictly necessary (AND Boolean rule), the mean of the abundance was calculated. The thickness of the lines is proportional to the abundance of the transcripts involved in the given reaction. Constructed in Escher (King et al., 2015).

In EPS metabolism, xanthan production genes were repressed in the *rpfCGH* mutant, indicating a positive regulation by QS (direct or indirect). Seven *gum* genes (*gumM*, *gumK*, *gumI*, *gumH*, *gumG*, *gumF*, and *gumD*) showed lower levels of expression in the *rpfCGH* mutant when compared with the wild-type strain. However, guml and gumBCEJ show smaller differences between the two strains. Figure 6 shows a comparison of xanthan production reactions between the two strains using gene expression levels.

Showing these changes on a metabolic map allows one to identify highly expressed reactions across metabolic pathways. Figure 2 shows the carbohydrate utilization pathways; these pathways were highly active at the transcriptional level in both strains. The most active reactions were fructose-bisphosphate aldolase and sedoheptulose 1, 7-bisphosphate D-glyceraldehyde-3-phosphate-lyase, which had an unusual abundance of transcripts (thousands of FPKMs compared to a median of 156 and 131 for the rest of the genes in both strains tested).

### Integration of Expression Profiles Into *Xpm* Metabolic Model Shows Xanthan, Purine, Pyrimidine, and Amino Acid Metabolism Are Altered by QS

The context-specific models constructed using iMAT are summarized in Table 1; the metabolic models can be downloaded from GitHub^5^. The models showed small differences in number of reactions, compounds, and genes. When the reactions between the two models were compared, only 32 and 26 reactions were unique for *Xpm* CIO151 EV and *Xpm* CIO151 Δ*rpfCGH*-EV, respectively (Supplementary Table S15). They shared most of their reactions (653; Supplementary Table S15). A flux balance analysis of the CSMs also gave the same predicted growth rate value of 0.0986 h^–1^ for both models. The experimental growth rate measured in Phi ϕ medium was similar for both strains (Supplementary Table S16 and Supplementary Figure S5). The pathway categories of the unique reactions in *Xpm* CIO151 EV included Entner–Doudoroff, pentose phosphate, glycerolipid, phosphatidylserine, pyruvate, and pyrimidine metabolism. The pathway categories of the unique reactions in *Xpm* CIO151 Δ*rpfCGH*-EV included fatty acid, fermentation, proline synthesis, and purine conversion.

**TABLE 1:** Summary of the context-specific models of metabolism for *Xpm* CIO151 EV and *Xpm* CIO151 Δ*rpfCGH*-EV.

	*Xpm* CIO151 EV	*Xpm* CIO151 Δ *rpfCGH-*EV
Reactions	685	679
Compounds	618	614
Genes	617	618

*The number of reactions, compounds, and genes for every model is shown.*

Surprisingly, although the genes related with the reactions of xanthan production were overexpressed in the *rpfGH* mutant with respect to the wild type (analysis with NOISeqBio), they were not excluded from the metabolic model of the mutant by iMAT. However, the abundances of transcripts for the majority of genes in the *gum* cluster were half for *Xpm* CIO151 Δ*rpfCGH*-EV when compared to *Xpm* CIO151 EV (Figure 6). Interestingly, the last step of xanthan transport performed by *gumL* and the cluster of *gumBCEJ* were only slightly underexpressed in *Xpm* CIO151 Δ*rpfCGH*-EV with respect to *Xpm* CIO151 EV.

**FIGURE 7:**
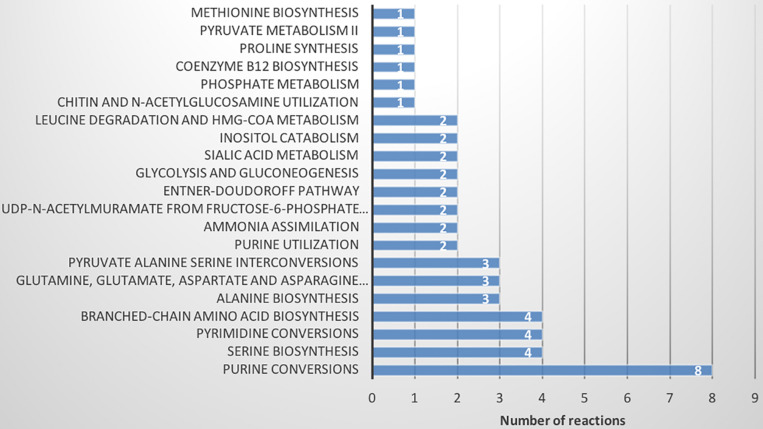
Metabolic pathways found in flux balance analysis that differentiate *Xpm* strains (*Xpm* CIO151 EV and *Xpm* CIO151 Δ*rpfCGH-EV*) and are influenced by quorum sensing. The number of reactions per pathway is shown.

Finally, FBA of each CSM was used to calculate the distribution of fluxes of each model. The fluxes of each reaction were grouped by hierarchical clustering (Supplementary Figure S6 and Supplementary Tables S17, S18). A total of 618 active reactions (at least in one condition) in FBA were used for the hierarchical clustering. We found six groups of reactions, three of which did not show a change between the CSMs (purple, orange and blue, 562 reactions; Supplementary Table S18). The blue group is characterized by a high proportion of low flux values. The other groups (green, red and brown) differentiated *Xpm* CIO151 EV from *Xpm* CIO151 Δ*rpfCGH*-EV (51 reactions). Figure 7 shows the pathways represented in the reactions that differentiate the two strains. The main pathways influenced by quorum sensing are purine, serine, pyrimidine, and amino acid metabolism. Again, the Entner–Doudoroff pathway is influenced by quorum sensing. Finally, the objective function of biomass plus xanthan did not separate the two strains; thus, the groups were instead determined by the strain.

## Discussion

In this study, the metabolic model of *Xpm* was reconstructed at the genome scale. The main features of this model were depicted and simulated *in silico*, especially those related with quorum sensing and xanthan production. Bacterial growth using an alternative objective function of biomass with xanthan was modeled, exposing the implications of this polymer in resource allocation processes. The dynamics of the mechanism of xanthan production and its connection to carbohydrate utilization pathways were reconstructed and modeled in *Xpm*, showing a trade-off between biomass and xanthan production. The model shed light onto the transport mechanism for several carbohydrate sources (e.g., sucrose, fructose, and mannose). Furthermore, data and analyses of differential expression assays of a quorum sensing mutant were used for understanding this bacterial mechanism in *Xpm*. The visualization of transcript abundance of these data on Escher maps helped to understand some differences at the metabolic level. These included the xanthan biosynthetic route, as well as carbohydrate and central metabolism, notably the balance of NAD(P)^+^. Furthermore, RNA-Seq data were used to construct and analyze the CSMs of the *Xpm* wild type and a mutant of quorum sensing. A flux balance analysis of these CSMs showed differences in the flux of the reactions for the two strains studied here. Some of the main metabolic pathways identified as altered by quorum sensing were amino acid and nitrogen metabolism, as well as fatty acid elongation. Finally, other important contributions to the metabolic modeling of *Xpm* were three manually curated maps of central carbohydrate metabolism, xanthan production, and amino acids that will serve as visualization tools for *Xpm* metabolism and that of other *Xanthomonas*.

In this study, the differences in the production of biomass with two scenarios (different objective functions) can be explained by the divergence of sugars into the xanthan route from the ED pathway. This could be related to the cycles of growth vs. generation of aggregates in the vascular system or at the epiphytic stages of the bacterium in the plant. When the bacterium is growing, xanthan production decreases, and when it is forming aggregates and colonies, the production of xanthan is increased in detriment of growth (Bren et al., 2016). This trade-off is in agreement with other models such as those for *Xcc* and *Ralstonia solanacearum*, which have used a similar approach to model pathogenicity, with a modified objective function (Edwards et al., 2001). Further pathogenicity and survivorship factors can be modeled in *Xpm* in the future using the same strategy. For example, the balance between transport and catabolism of carbohydrate sources and organic acids and the impact of this balance in xanthan production can be researched using our model with the objective function of xanthan biomass, simulating different stages along the infection.

Twelve genes implicated in the reactions of the xanthan pathway were overexpressed after the alteration of the QS proccess. Some of them are important for processes related with QS and pathogenicity. *gumD* was reported to be important for pathogenicity, EPS production, and epiphytic survival of *Xpm* (Kemp et al., 2004), and mutants of *gumK* and *gumF* reduced water soaking and produced thicker lesions in *X. citri* pv. *citri* (Kang et al., 1999; Newman et al., 1994; Peyraud et al., 2016; Vojnov et al., 2001). Interestingly, we found that, among the genes in this route, the *gumL* and the *gumBCEJ* clusters were the least affected in the QS mutant and, therefore, are the least altered by the knockout of RpfCGH. These genes encode enzymes responsible for the final steps of polymerization and the transport of xanthan, and proteins encoded by *gumBCEJ* are proposed to be anchored to the inner membrane and the periplasm (Peyraud et al., 2016; Schatschneider et al., 2013; Vorhölter et al., 2008). Two hypotheses can explain this result: (1) Transporters are regulated by other operons of genes different from RpfCGH but still related to the regulation of QS or (2) there is a necessity to maintain the membrane transporters to avoid the accumulation of xanthan due to its toxicity. Mutations in *gumBCE* were lethal for *Xcc*, presumably because the lipid precursors produced as intermediaries in the production of xanthan were toxic by accumulation (Yan and Wang, 2012). Therefore, it is hypothesized that *Xanthomonas* has evolved to maintain a constant expression of the transporter, despite the reduction of xanthan production, to avoid the toxicity of xanthan intermediates by accumulation.

Differences at the transcriptional level were captured by the FBA of the CSMs for the Entner–Doudoroff pathway, which is connected with xanthan production. However, other differences were not captured in the CSMs, including the other carbohydrate utilization pathways connected with xanthan production. Although the algorithm used to construct the CSMs (iMAT) maximizes the number of reactions to be included in each CSM based on the transcript levels, the method does not restrict the flux levels based on these data in order to improve the metabolic simulations in the FBA. In the future, fluxomics data (Gomes and Simões, 2012; Lewis et al., 2012) and other methods that integrate these kinds of data into genome-scale metabolic models can be used to capture the real differences in phenotype and improve metabolic predictions, as has been shown before in *Xcc* (Katzen et al., 1998; Vojnov et al., 2001). Another limitation of the iMAT approach is that iMAT does not impose constraints in the growth rate when RNA-seq data are integrated into the metabolic model. Other methods that impose restrictions in the objective function as GIMME or MADE (Becker and Palsson, 2008; Jensen and Papin, 2011) or including experimentally measured constraints in the CSM (Schatschneider et al., 2014) can be used. However, for the two strains tested here, this is irrelevant since the growth rate of the strains in the laboratory is statistically similar (Supplementary Figure S5 and Supplementary Table S16).

Construct context-specific models showed remarkable differences between the two strains at the level of amino acid and nitrogen metabolism when FBA was performed and compared by hierarchical clustering (12 amino-acid-related pathways). This highlights the alteration of amino acid metabolism by quorum sensing. In *Xcc*, the DSF family of QS signals requires branched amino acid precursors (Becker and Palsson, 2008; Jensen and Papin, 2011), whose synthesis were found to be differentially active in FBA for the two strains in our study. Thus, the role of the other reactions related with amino acid metabolism should be researched further. In *Xcc*, the fatty acid elongation cycle was also found to be necessary for DSF production (Machado and Herrgård, 2014). We found two reactions unique to RpfCGH mutants in the CSM related to fatty acid biosynthesis: an acetyl-CoA carboxylase (biotin-dependent) and a biotin carboxylase. The enzymes are part of a conserved system in bacteria, the fatty acid synthase II (FAS II) complex.

Several enzymes related with reactions that influence the balance of NAD(P)^+^ were differentially expressed, including reactions for carbohydrate metabolism and amino acid biosynthesis. NAD(P)^+^ transhydrogenase, an enzyme reported as the major source of NADPH in *E. coli* (Zhou et al., 2015), was downregulated in the QS mutant. NAD(P)^+^ transhydrogenase reaction regulates the level of NADPH in the cell through the transfer of hydride ion and aids in removing reactive species (Davenport et al., 2015; Goo et al., 2017; Uzureau et al., 2010). In the case of carbohydrate metabolism, five reactions related with NAD metabolism were activated in the *rpfCGH* mutant with respect to the wild type (formate dehydrogenase, two reactions of glyceraldehyde-3-phosphate dehydrogenase, methylmalonate-semialdehyde dehydrogenase, and isocitrate dehydrogenase), some of them previously reported as regulated by QS in other genera of bacteria (Zhou et al., 2015). Quorum sensing also regulates pentose phosphate, a carbohydrate utilization pathway; it has been suggested that the increased level of NADPH in QS mutants in other bacteria could be related with the pentose phosphate pathway (Sauer et al., 2003). In our analyses, only one reaction was differentially expressed and related with both the pentose phosphate pathway and the NAD metabolism—the phosphogluconate dehydrogenase; in addition, we found that carbohydrate sources connected with carbohydrate utilization pathways are also altered in the *rpfCGH* mutant. Therefore, it can be proposed that QS in *Xpm* orchestrates a balance between carbohydrate and NAD metabolism by the pentose phosphate pathway and the transhydrogenase activity. Thus, levels of NADPH can be influenced by both the downregulation of NAD(P)^+^ transhydrogenase and the carbohydrate pathway reactions in the QS mutant.

Several genes related with carbohydrate metabolism were found to be differentially expressed by *Xpm*, such as transport of fumarate, citrate, trehalose, which were suppressed, and D-mannose (via PEP:Pyr PTS), which was induced in the QS mutant. Trehalose is connected to the carbohydrate utilization pathways through the D-glucose-6-phosphate, and it can influence xanthan metabolism. The reactions affected in the trehalose metabolism included the transformations of maltose to trehalose and of trehalose-6-phosphate to glucose-6-phosphate. In contrast, fumarate and citrate are directly connected to the central metabolism. It has been proposed that quorum sensing regulates carbohydrate uptake and utilization pathways in bacteria and slows down central metabolism, functioning as a metabolic brake (García-Contreras et al., 2015). Our results are in agreement with this proposal for *Xpm*. These examples show how some important enzymes related with carbohydrate metabolism are under- and over-expressed in the *rpCGH* mutant, supporting the hypothesis of carbohydrate metabolism alteration by QS system.

The modeling of an objective function that includes xanthan production is useful to understand its connection with central metabolism, e.g., pyruvate metabolism. We found two reactions related to pyruvate metabolism and pyruvate, alanine, and serine interconversions that differentiate the two strains in CSM. Pyruvate dehydrogenase has been reported and considerably studied in *X. campestris* due to its importance in xanthan production (An et al., 2014; Davenport et al., 2015; Goo et al., 2015). Pyruvate dehydrogenase is a complex of three enzymes that catalyze the reaction of interconversion of pyruvate to acetyl-coA within the central metabolism. Thus, quorum sensing can alter several reactions in the central metabolism of *Xpm* as well as those related with xanthan biosynthesis.

We propose previously unknown mechanisms of transport in *Xpm* for some metabolized carbohydrate sources derived of the structural analysis of the metabolic model and its manual curation with the Escher tool. For example, we propose a mechanism for the transport of mannose and fructose in *Xpm* using PEP:Pyr PTS transport system coupled with fructose 6-phosphate. This transport mechanism has been reported to transport other carbohydrate sources in *Xcc* such as sucrose and mannitol (An et al., 2014; Davenport et al., 2015; Goo et al., 2015). Sucrose has been reported to be used as a carbohydrate source in *Xpm* (Iliev and Ivanova, 2002); however, there is evidence against this transport system for *Xanthomonas citri* pv. *glycines* (previously named *X. axonopodis* pv. *glycines*) (de Crecy-Lagard et al., 1995). The evidence in *Xcg* favors transport into the cell without lysis of sucrose and subsequent hydrolysis at the intracellular level. The enzymes required for internal hydrolysis are present in the *Xpm* model. In addition, there is a gene in *Xpm* with a high identity with the *suc1* transporter from *X. citri* pv. *citri* (previously named *X. axonopodis* pv. *citri*) (BLAST, e-value > 1E6, 100% coverage). This transporter was first reported in *X. phaseoli* (Van den Mooter et al., 1987), and it is present in other *Xanthomonas*. All these evidences support the hypothesis of the same mechanism in *Xpm* for the import of sucrose with subsequent intracellular hydrolysis.

It is important to highlight some points related to the differential expression assays for the RpfCGH mutant of *Xpm*. RpfCGH is involved in the upregulation of several genes within the Rpf-dependent QS pathway; a global expression pattern that agrees with previous results published by Guo (Kim et al., 2004) for single mutants of rpfC and rpfG in *Xanthomonas citri* pv. *citri*. Furthermore, the genes rpfC, rpfG, and 11 *gum* genes involved in extracellular polysaccharide production and biofilm formation were always detected as downregulated in the mutant strain. This is in agreement with previous reports for these genes in *Xanthomonas* (Hochster and Katznelson, 1958). Also, the Fisher enrichment analysis for the DEGs of the RpfCGH mutant showed altered molecular functions of Xpm CIO151, previously reported as influenced by the QS regulatory network (Guo et al., 2012; He et al., 2006). These include bacterial-type flagellar motility, chemotaxis, signal transducer activity, and oxidoreductase activity. Furthermore, genes involved in the T3SS, mainly hrcS and hpa3, were affected, suggesting a positive regulation of T3SS by QS as has been shown in other systems (He et al., 2006).

## Conclusion and Perspectives

We present here the metabolic model of *Xpm*. Some important pathways related to quorum sensing, pathogenicity, and defense were incorporated and studied using FBA. Alternative objective function modeling shows a trade-off and resource allocation between the growth and the biosynthesis of xanthan, an important pathogenicity and survival factor of *Xpm*. Hierarchical clustering showed differentiation in groups of reactions in the two strains studied when simulated by FBA of the CSMs. Part of these groups is related to the differences in QS. For example, carbohydrate sources such as fumarate, citrate, trehalose, and D-mannose transported *via* PEP:Pyr PTS were differentially expressed between the QS mutant strain and WT. Some of them are connected with central metabolism and an additional one to carbohydrate utilization pathways. Importantly, NAD(P)^+^ transhydrogenase had a lower expression in the QS mutant, affecting the NADPH levels in a DSF-dependent manner. All the genes and reactions related to the pathways studied here can be used as a starting point for gaining new insights in *Xanthomonas* metabolism.

The metabolic model reconstructed here and the mathematical modeling performed using pathogenicity factors such as xanthan, along with the integration of more information from experiments, both *in vitro* and *in planta*, will expand the knowledge of the interaction between *Xpm* and cassava. This work is just the first step to a fully comprehensive understanding of the metabolism of *Xpm* as a plant pathogen through a systems biology approach. Our work poses more questions in the metabolic status of the bacterium before and during *in planta* growth, which can be addressed using our metabolic model and experimentation. For example, how does *Xpm* balance importation and metabolism of organic acids to produce xanthan and multiply in the plant? What is the effect of pyruvate dehydrogenase in the production of xanthan in *Xpm*? It would be important to improve and refine the metabolic model and to integrate other types of omics data and physiological information, including *in planta* studies. We also expect that this metabolic model will serve to improve knowledge of other plant pathogens belonging to the *Xanthomonas* genus.

## Data Availability Statement

The datasets generated for this study can be found in the NCBI under the projectID PRJNA598165 and SRA codes SRX7570863–SRX7570866. All related code, models and maps can be downloaded from GitHub: https://github.com/davidoctaviobotero/Xpm_metabolic_model – a detailed list is available in Supplementary Data Sheet S2.

## Author Contributions

AB, SR, AG, and DB designed the study. AR advised the metabolic analyses. AB, ARC, MR, and VB-G designed and performed the experimental procedures and the analyses for RNA-seq and elaborated the first draft of the transcriptional analyses. AG, BP, and JM designed and supported COBRA approach for *Xpm* and advised all the computational analyses. MJR designed, performed, and analyzed the xanthan maps and the computational simulations. DB designed and performed the computational analyses, models, and maps, integrated the metabolic model and the experimental results, and elaborated the first draft of the manuscript and the corrections. All authors contributed to writing the manuscript and approved its final version.

## Conflict of Interest

The authors declare that the research was conducted in the absence of any commercial or financial relationships that could be construed as a potential conflict of interest.

## Footnotes

1 https://iant.toulouse.inra.fr/bacteria/annotation/cgi/xanmn/xanmn.cgi

2 http://hannonlab.cshl.edu/fastx_toolkit/

3 https://github.com/davidoctaviobotero/Xpm_metabolic_model/tree/master/Maps

4 https://github.com/davidoctaviobotero/Xpm_metabolic_model/blob/master/Maps/Xpm-Aminoacid_map.json

5 https://github.com/davidoctaviobotero/Xpm_metabolic_model/tree/master/Models

## Abbreviations

CBB: cassava bacterial blight
CFU: colony-forming units
COBRA: Constraint-Based Reconstruction and Analysis
CSMs: context-specific models
DEGs: differentially expressed genes
DSF: diffusible signal factor
DXPS: 1-deoxy-D-xylulose 5-phosphate synthase
EC: enzyme commission numbers
ED: Entner–Doudoroff pathway
EMP: Embden–Meyerhof–Parnas
EPS: exopolysaccharide
FAS II: fatty acid synthase II
FBA react: fructose-bisphosphate aldolase reaction
FBA: flux balance analysis
FBA3, sedoheptulose 1: 7-bisphosphate D-glyceraldehyde -3 phosphate-lyase
FPKM: fragments per kilobase of transcript per million mapped reads
GO: Gene Ontology
HSP: high-scoring segment pair
MAN6PI: mannose-6-phosphate isomerase
NAD(P)^+^: nicotinmide adenine dinucleotide phosphate
PEP: phosphoenolpyruvate
PEP:Pyr PTS: (PEP)-dependent phosphotransferase system
PGMT: phosphoglucomutase
PP: pentose-phosphate
PTS: (PEP)-dependent phosphotransferase system
QS: quorum sensing
RBH: BLAST reciprocal best hit; reversible transportation (pyruvate to PEP)
T3SS: type 3 secretion system
*Xcc*: *Xanthomonas campestris* pv. *campestris*
*Xci*: *Xanthomonas citri*
*Xpm*: *Xanthomonas phaseoli* pv. *manihotis.*
