# Supplementary material for: Genome-Scale Metabolic Model of Xanthomonas phaseoli pv. manihotis: An Approach to Elucidate Pathogenicity at the Metabolic Level
Source: Front Genet. 2020 Aug 11;11:837. doi: 10.3389/fgene.2020.00837 (PMC7432306; doi:10.3389/fgene.2020.00837)
Supplement: Supplementary file 1 [file Data_Sheet_1.docx]

**Supplementary Data Sheet 1:** Meta analysis with the literature reviewed in the manual curation process of the metabolic network.

| **Name** | **Notes** | **References** |
| --- | --- | --- |
| Mannitol | Mannitol transport system deleted, Evidence found in literature that is not metabolized (also ⅕ genes only found in the model). | (Ogunjobi et al., 2007)⁠ |
| Sucrose | Kim, 2004 has evidence against PTS transport system for *Xag* for the case of sucrose.  Evidence support transport without lysis of sucrose and hydrolysis at the intracellular level.  Enzymes required for internal hydrolysis present in the model.  Blast of the transporter suc1 of Xac hit (100%) to Xam protein previously annotated as glycoside-cation-symporter.  Add reaction to the model. Delete the PTS-sucrose reaction of the model. | (de Crecy-Lagard et al., 1995; H.-S. Kim, Park, Heu, & Jung, 2004)⁠ |
| Citrate | Oxidative metabolism reported in *Xpm*. | (Ogunjobi et al., 2007; Zimaro et al., 2011)⁠ |
| Maltose | Oxidative metabolism reported in *Xpm*. | (Ogunjobi et al., 2007)⁠ |
| Arabinose | Oxidative metabolism reported in *Xpm*. | (Ogunjobi et al., 2007)⁠ |
| Trehalose | Oxidative metabolism reported in Xpm. | (Mwangi et al., 1999; Ogunjobi et al., 2007)⁠ |
| Cellobiose | Oxidative metabolism reported in *Xpm*. | (Mwangi et al., 1999; Ogunjobi et al., 2007)⁠⁠ |
| Fructose | Oxidative metabolism reported in *Xpm*. | (de Crecy-Lagard et al., 1995; Ogunjobi et al., 2007)⁠ |
| Mannose | Oxidative metabolism reported in *Xpm*. | (de Crecy-Lagard, Binet, & Danchin, 1995)⁠ |
| Xylose | Oxidative metabolism reported in *Xpm*. | (Déjean et al., 2013; Ogunjobi, Fagade, & Dixon, 2007; Zhang & Chen, 2010)⁠⁠ |
| Galactose | Oxidative metabolism reported in *Xpm*. | (Ogunjobi et al., 2007)⁠ |
| Xanthan |  | (Zhang & Chen, 2010)⁠ |
| N-acetyl-D-glucosamine (*acgam*) | N-Glycans are degraded in Xcc. | (Dupoiron et al., 2015)⁠ |
| Acetaldehyde, Formate, Pyruvate | Exportation reactions added |  |
| Entner–Doudoroff (ED) | First report was done in *X. phaseoli* (Hochster & Katznelson, 1958) and present in other *Xanthomonas* .  Clues of “preferentially use of organic acids rather than sugars and secretion of exopolysaccharides” in some *Xanthomonas* (Fuhrer, 2005). | (Fuhrer, Fischer, & Sauer, 2005; Hochster & Katznelson, 1958; Madsen & Hochster, 1959)⁠ |
| Pyruvate dehydrogenase (pyr-accoa) | Reported and deeply studied in *X. campestris* due to its importance in the production of Xanthan (virulence factor and industrial product), (Iliev & Ivanova, 2002).  Pyruvate dehydrogenase is a complex of three enzymes. The three enzymes have significant blast in the genome of *Xpm* and were previously annotated.  Correction of annotation and addition of the reaction. | (Iliev & Ivanova, 2002)⁠ |
| Acetate | Phosphotransacetylase and acetate kinase are not present. Instead, Acetyl-CoA synthetase (ACS) is present.  There is not transport and exchange reactions associated with acetate. However, the transport has been reported in *Xcc*, (Tang, 2005; Moser, 2014).  The *E. col*i transporter hit poorly *Xpm* proteins.  But, the acetate cation (Na+) symporter have been annotated in the *Xpm* genome previously. Add ACt4pp (BiGG) to the model. | (Moser, Aktas, & Narberhaus, 2014; Tang et al., 2005)⁠ |
| Ethanol metabolism (degradation) | Ethanol resistance has been characterized in Xcc (Bordes, 2011) regulated by the sigma-E factor.  Although there is no specific experimental evidence of the ethanol degradation pathway, there are reports at the genome level for other *Xanthomonas* of that pathway.  Transport by diffusion of ethanol (aqueous porins).  No evidence for acetaldehyde transport  No reaction for ACALD: accoa <--> acald, no evidence found. Deleted. | (Bordes et al., 2011)⁠ |
| Ethanolamine | Metabolism of ethanolamine included, evidence in LPS as virulence factor in *Xac*.  Transporter appears as an orphan reaction in DB.  *Xanthomonas* not growth up in ethanolamine as carbon source?  How to reconcile these two results? | (Casabuono, Petrocelli, Ottado, Orellano, & Couto, 2011)⁠ |
| Lactate | Absent: D-lactate dehydrogenase and D-lactate transport via proton symport  Present: L-Lactate dehydrogenase (LDH-L)  Evidence:  LDH-L is reported in Uniprot for Xcc.  Reported as recombinant protein (MyBiosource).  Lactate dehydrogenase has been isolated and its enzymatic activity has been measured in Xcc (Sitkin, 2003).  Non-transport reaction  Solving then within pyruvate metabolism map | (Sitkin, Tsfasman, Stepnaya, & Kulaev, 2003)⁠ |
| 2-oxoglutarate reversible transport via symport | The reaction has been reported in Xoo. Surprisingly, a similar transporter is also secreted and anchored into the plant membrane for extraction of *akg*. | (Guo et al., 2012)⁠ |
| L-Glutamine ABC transporter | Improving annotation: unless non transport literature evidence was found, Blasting of the Glutamine transport ATP-binding protein of *E. coli* against *Xpm*, significantly hit several proteins previously annotated as ABC amino acid transporters.  High levels of glutamine are used for *Arabidopsis* plants as mechanism of defense. This high levels activate a di-GMP signaling system of defense in *Xcc*, (an Arabidopsis pathogen). This point could be researched in *Xpm* later... |  |
| Succinate-fumarate interconversion | The succinate dehydrogenase complex (Xac) is an alternative pathway in Xam model (all the reactions are present in the Xam model):  FRD7. Irreversible (correction of reversibility according to E. coli).  FRD: Succ to Fum (Fad:Fadh2). Correct reversibility according to Zimaro et. al., 2013.  SUCD4: Fadh2:Fad | (Zimaro et al., 2013)⁠ |
| Oxaloacetate interconversion Phosphoenolpyruvate | PPCK: *Xcc* have only the malic enzyme-PpsA route in gluconeogenesis. Deleted.  PPC reversible, corrected, irreversible  Malate dehydrogenase (oxaloacetate decarboxylating) (NADP+), malic enzyme.  PEPCK: using gtp, irreversible, possible hypothesis to evaluate later?  MNXR71850: using itp, reversible, possible hypothesis to evaluate later? |  |
|  | Change Stoichiometry. Cytochrome oxidase  CYTBD - CYTBO3 |  |
|  | Change Stoichiometry. NADH dehydrogenase  NADH16 - NADH6 |  |
|  | SUCCT3 – SUCCt2b, Change of reversibility |  |
| Growth Media | Minimal media and full media for *Xanthomonas* | (Cohn et al., 2014; Mwangi et al., 1999; Stoyanova, Vancheva, Moncheva, & Bogatzevska, 2014; Zimaro et al., 2013)⁠ |
| 2-keto-3-deoxygluconate | ED modified pathway | (S. Kim & Lee, 2006)⁠ |
| 4-Hydroxybenzoate | Pyruvate: Citric acid cycle and Oxidative phosphorylation | (Crawford, 1975)⁠ |
| Adenosine | Adenosine savage pathway. Important for EPS, cell motility and virulence. ATP synthesized *de novo* by this reason the kinase enzyme non necessary for growth (non-minimal media requirement). | (Lu et al., 2009)⁠ |
| Aminoethanol | It is reported a non-carbon source for *Xanthomonas* | (Swings & Civerolo, 1993)⁠ |
| BET(Betaine) | Osmoprotectant synthesized form coline. Derivative aminoacid in plants, glycine betaine. Intermediate in the catabolic pathway of choline and its precursors. | (Mori, Yoshida, & Kitamoto, 1992)⁠ |
| Cu2 | Copper resitance | (Voloudakis, Reignier, & Cooksey, 2005)⁠ |
| D-Galacturonate | Celullose, plant cell wall. | (Payne & Carlson, 1957)⁠ |
| Glucuronate | Xanthan compound; Hidrolysis of starch | (Payne & Carlson, 1957)⁠ |
| Hypoxanthine |  | (Yuan, Wang, Sun, Wu, & Qian, 2013)⁠ |
| L_Arginine |  | (Swings & Civerolo, 1993)⁠ |
| L_Proline |  | (Swings & Civerolo, 1993)⁠ |
| L_Aspartate |  | (Swings & Civerolo, 1993)⁠ |
| L_Lysine |  | (Swings & Civerolo, 1993)⁠ |
| L_Methionine |  | (Swings & Civerolo, 1993)⁠ |
| L_Glutamate |  | (Rojas, Nishidomi, Nepomuceno, Oshiro, & de Cassia Café Ferreira, 2013)⁠ |
| L_Tryptophan |  | (Swings & Civerolo, 1993)⁠ |
| NH_3_ |  | (Swings & Civerolo, 1993)⁠ |
| Nicotinamide ribonucleotide | NAD savage pathway | (Gazzaniga, Stebbins, Chang, McPeek, & Brenner, 2009)⁠ |
| Palmitate | Saturated fatty acid. Pathogenicity factor. | (Bishop, Kim, & El Zoeiby, 2005)⁠ |
| Succinate | Citric acid cycle: Important for induction of T3SS in bacterium. Krebs, Tricarboxylic Acid cycle TCA. |  |
| Gene cluster gumBCDEFGHIJKLM | 16 Kb cluster that are expressed as an operon of a promoter upstream of the first gene in this case is gumB. Identified for *Xanthomonas campestris* pv. *campestris*, which is why an alignment of the gene sequence was performed with the Xpm genome with an 85.96% identity, a score of 13.955 and an e-value of 0 | (Schatschneider et al., 2013; Vorhölter et al., 2008)⁠ |
| gene gumD | The presence of the gumD gene encoding the enzyme undecaprenyl phosphate glucose phosphotransferase involved in the xanthan formation process was identified. The presence in the *Xpm* genome was identified by a Blastn alignment with 99.07% identity, a score of 7,350 and an e-value of 0 with *Xanthomonas campestris* pv. *campestris* genome | (Vorhölter et al., 2008)⁠ |
| gene gumM | The presence of the gumM gene encoding the beta-1,4-glucosyltransferase enzyme involved in the xanthan formation process was identified. If you corroborated the presence in the Xpm genome through a tBlastn type alignment with 99.57% identity, a score of 1,210 and an e-value of 3.50247 e-123 with *Xanthomonas campestris* pv. *campestris* genome | (Vorhölter et al., 2008) |
| gene gumH | The presence of the gumH gene that encodes the enzyme alpha-1,3-mannosyltransferase involved in the xanthan formation process was identified. The presence in the Xpm genome was corroborated by a tBlastn type alignment with 91.05% identity, a score of 1,786 and an e-value of 3,12024 e-184 with *Xanthomonas campestris* pv. *campestris* genome | (Katzen et al., 1998)⁠ |
| gene gumK | The presence of the gumK gene encoding the enzyme 2-beta-glucuronyltransferase involved in the xanthan formation process was identified. The presence in the Xpm genome was corroborated by a tBlastn type alignment with 92.2% identity, a score of 1,435 and an e-value of 6,34862 e-147 with *Xanthomonas campestris* pv. *campestris* genome | (Barreras, Abdian, & Ielpi, 2004)⁠ |
| gene gumI | The presence of the gumK gene encoding the beta-1,4-mannosyltransferase enzyme involved in the xanthan formation process was identified. The presence in the Xpm genome was corroborated by a tBlastn type alignment with 85.39% identity, a score of 1,561 and an e-value of 2,19767e-160 with *Xanthomonas campestris* pv. *campestris* genome | (Vorhölter et al., 2008) |
| gene gumF | The presence of the gumF gene that encodes the enzyme with acyltransferase activity involved in the xanthan formation process was identified. The presence in the Xpm genome was corroborated by a tBlastn type alignment with 78.79% identity, a score of 1536 and an e-value of 4.87597 e-157 with *Xanthomonas campestris* pv. *campestris* genome | (Vorhölter et al., 2008) |
| gene gumL | The presence of the gumL gene that encodes the enzyme pyruvyltransferase involved in the xanthan formation process was identified. The presence in the Xpm genome was corroborated by a tBlastn type alignment with 90.91% identity, a score of 1,305 and an e-value of 2.98785 e-133 with *Xanthomonas campestris* pv. *campestris* genome | (Vorhölter et al., 2008) |
| gene gumG | The presence of the gumG gene encoding the enzyme with acyltransferase activity involved in the xanthan formation process was identified. The presence in the Xpm genome was corroborated by a tBlastn type alignment with 67.27% identity, a score of 1,180 and an e-value of 4,38979 e-123 with *Xanthomonas campestris* pv. *campestris* genome | (Vorhölter et al., 2008) |
| gene gumB | The presence of the gumB gene encoding an enzyme involved in the xanthan formation process was identified. The presence in the Xpm genome was corroborated by a tBlastn type alignment with 80.89% identity, a score of 1,095 and an e-value of 2.13405 e-59 with *Xanthomonas campestris* pv. *campestris* genome | Uniprot access number Q93A84-1 , published by Ielmini M.V., Katzen F., Lelpi L. in 2001 |
| gene gumC | The presence of the gumC gene that encodes the enzyme involved in the xanthan formation process was identified. The presence in the Xpm genome was corroborated by a tBlastn type alignment with 67.27% identity, a score of 1,180 and an e-value of 4,38979 e-123 with *Xanthomonas campestris* pv. *campestris* genome | (Vorhölter et al., 2008) |
| gene gumE | The presence of the gumE gene that encodes the putative xanthan polymerase enzyme involved in the xanthan formation process was identified. The presence in the Xpm genome was corroborated by a tBlastn type alignment with 86.06% identity, a score of 5,110 and an e-value of 3,62589e-295 with *Xanthomonas campestris* pv. *campestris* genome | (Vorhölter et al., 2008) |
| Gene gumJ | The presence of the gumJ gene encoding an enzyme involved in the xanthan formation process was identified. The presence in the Xpm genome was corroborated by a tBlastn type alignment with 89.31% identity, a score of 2,299 and an e-value of 1,032143 e-238 with *Xanthomonas campestris* pv. *campestris* genome | (Da Silva et al., 2002)⁠ |
| Biosynthesis of amino acids – Pathway | The pathway has been built from the reactions involved in the initial reconstruction and that were identified in the reference map published in the KEGG database. This map served as a guide for the reconstruction of the amino acid biosynthesis pathway | (Ogata et al., 1999)⁠ |

**References**

Barreras, M., Abdian, P. L., & Ielpi, L. (2004). Functional characterization of GumK, a membrane-associated β-glucuronosyltransferase from *Xanthomonas campestris* required for xanthan polysaccharide synthesis. *Glycobiology*. https://doi.org/10.1093/glycob/cwh056

Bishop, R. E., Kim, S.-H., & El Zoeiby, A. (2005). Role of lipid A palmitoylation in bacterial pathogenesis. *Journal of Endotoxin Research*, *11*(3), 174–180. https://doi.org/10.1179/096805105X35242

Bordes, P., Lavatine, L., Phok, K., Barriot, R., Boulanger, A., Castanié-Cornet, M. P., … Gutierrez, C. (2011). Insights into the extracytoplasmic stress response of *Xanthomonas campestris* pv. *campestris*: Role and regulation of σE-dependent activity. *Journal of Bacteriology*, *193*(1), 246–264. https://doi.org/10.1128/JB.00884-10

Casabuono, A., Petrocelli, S., Ottado, J., Orellano, E. G., & Couto, A. S. (2011). Structural analysis and involvement in plant innate immunity of *Xanthomonas axonopodis* pv. *citri* lipopolysaccharide. *Journal of Biological Chemistry*, *286*(29), 25628–25643. https://doi.org/10.1074/jbc.M110.186049

Cohn, M., Bart, R. S., Shybut, M., Dahlbeck, D., Gomez, M., Morbitzer, R., … Staskawicz, B. J. (2014). *Xanthomonas axonopodis* Virulence Is Promoted by a Transcription Activator-Like Effector–Mediated Induction of a SWEET Sugar Transporter in Cassava. *Molecular Plant-Microbe Interactions*, *27*(11), 1186–1198. https://doi.org/10.1094/MPMI-06-14-0161-R

Crawford, R. L. (1975). Degradation of 3-hydroxybenzoate by bacteria of the genus *Bacillus*. *Applied Microbiology*, *30*(3), 439–444. Retrieved from http://www.ncbi.nlm.nih.gov/pubmed/810087

Da Silva, A. C. R., Ferro, J. A., Reinach, F. C., Farah, C. S., Furlan, L. R., Quaggio, R. B., … Kitajima, J. P. (2002). Comparison of the genomes of two *Xanthomonas* pathogens with differing host specificities. *Nature*. https://doi.org/10.1038/417459a

de Crecy-Lagard, V., Binet, M., & Danchin, A. (1995). Fructose phosphotransferase system of *Xanthomonas campestris* pv. *campestris*: characterization of the fruB gene. *Microbiology*, *141*(9), 2253–2260. https://doi.org/10.1099/13500872-141-9-2253

Déjean, G., Blanvillain-Baufumé, S., Boulanger, A., Darrasse, A., de Bernonville, T. D., Girard, A.-L., … Arlat, M. (2013). The xylan utilization system of the plant pathogen *Xanthomonas campestris* pv. *campestris* controls epiphytic life and reveals common features with oligotrophic bacteria and animal gut symbionts. *New Phytologist*, *198*(3), 899–915. https://doi.org/10.1111/nph.12187

Dupoiron, S., Zischek, C., Ligat, L., Carbonne, J., Boulanger, A., Dugé De Bernonville, T., … Albenne, C. (2015). The N-Glycan cluster from *Xanthomonas campestris* pv. *campestris*: A toolbox for sequential plant N-Glycan processing a toolbox for sequential plant N-Glycan processing. *Journal of Biological Chemistry*, *290*(10), 6022–6036. https://doi.org/10.1074/jbc.M114.624593

Fuhrer, T., Fischer, E., & Sauer, U. (2005). Experimental identification and quantification of glucose metabolism in seven bacterial species. *Society*, *187*(5), 1581–1590. https://doi.org/10.1128/JB.187.5.1581

Gazzaniga, F., Stebbins, R., Chang, S. Z., McPeek, M. A., & Brenner, C. (2009). Microbial NAD metabolism: lessons from comparative genomics. *Microbiology and Molecular Biology Reviews : MMBR*, *73*(3), 529–541, Table of Contents. https://doi.org/10.1128/MMBR.00042-08

Guo, W., Cai, L. L., Zou, H. S., Ma, W. X., Liu, X. L., Zou, L. F., … Chen, G. Y. (2012). Ketoglutarate transport protein KgtP is secreted through the type III secretion system and contributes to virulence in *Xanthomonas oryzae* pv. *oryzae*. *Applied and Environmental Microbiology*. https://doi.org/10.1128/AEM.07997-11

Hochster, R. M., & Katznelson, H. (1958). On the mechanism of glucose-6-phosphate oxidation in cell-free extracts of *Xanthomonas phaseoli* (xp8). *Biochemistry and Cell Biology*, *36*(7), 669–689. https://doi.org/10.1139/o58-074

Iliev, I., & Ivanova, I. (2002). Activity profiles of primary metabolism enzymes in *Xanthomonas campestris* strains during xanthan production. *Biotechnology and Biotechnological Equipment*, *16*(1), 77–82. https://doi.org/10.1080/13102818.2002.10819159

Katzen, F., Ferreiro, D. U., Oddo, C. G., Ielmini, M. V, Becker, A., Pühler, A., & Ielpi, L. (1998). *Xanthomonas campestris* pv. *campestris* gum mutants: effects on xanthan biosynthesis and plant virulence. *Journal of Bacteriology*, *180*(7), 1607–1617.

Kim, H.-S., Park, H.-J., Heu, S., & Jung, J. (2004). Molecular and functional characterization of a unique sucrose hydrolase from *Xanthomonas axonopodis* pv. *glycines*. *Journal of Bacteriology*, *186*(2), 411–418. https://doi.org/10.1128/JB.186.2.411

Kim, S., & Lee, S. B. (2006). Characterization of Sulfolobus solfataricus 2-Keto-3-deoxy- D -gluconate Kinase in the Modified Entner-Doudoroff Pathway. *Bioscience, Biotechnology, and Biochemistry*, *70*(6), 1308–1316. https://doi.org/10.1271/bbb.50566

Lu, G.-T., Tang, Y.-Q., Li, C.-Y., Li, R.-F., An, S.-Q., Feng, J.-X., … Tang, J.-L. (2009). An adenosine kinase exists in *Xanthomonas campestris* pathovar *campestris* and is involved in extracellular polysaccharide production, cell motility, and virulence. *Journal of Bacteriology*, *191*(11), 3639–3648. https://doi.org/10.1128/JB.00009-09

Madsen, N. B., & Hochster, R. M. (1959). The Tricarboxylic Acid and Glyoxilate Cycles in *Xanthomonas phaseoli* (XP8). *Canadian Journal of Microbiology*, *5*(1), 1–8. https://doi.org/10.1139/m59-001

Mori, N., Yoshida, N., & Kitamoto, Y. (1992). Purification and properties of betaine aldehyde dehydrogenase from *Xanthomonas translucens*. *Journal of Fermentation and Bioengineering*, *73*(5), 352–356. https://doi.org/10.1016/0922-338X(92)90277-2

Moser, R., Aktas, M., & Narberhaus, F. (2014). Phosphatidylcholine biosynthesis in *Xanthomonas campestris* via a yeast-like acylation pathway. *Molecular Microbiology*, *91*(4), 736–750. https://doi.org/10.1111/mmi.12492

Mwangi, M., Mwebaze, M., Bandyopadhyay, R., Aritua, V., Eden-Green, S., Tushemereirwe, W., … Rudolph, K. (1999). Development of a New Semiselective Medium for Isolating *Xanthomonas campestris* pv. *manihotis* from Plant Material and Soil. *Plant Pathology*, *89*(3), 591–597. https://doi.org/10.1094/PHYTO.1999.89.7.591

Ogata, H., Goto, S., Sato, K., Fujibuchi, W., Bono, H., & Kanehisa, M. (1999). KEGG: Kyoto encyclopedia of genes and genomes. *Nucleic Acids Research*, Vol. 27, pp. 29–34. https://doi.org/10.1093/nar/27.1.29

Ogunjobi, a. a., Fagade, O. E., & Dixon, A. G. O. (2007). Physiological studies on *Xanthomonas axonopodis* pv. *manihotis* (*Xam*) strains isolated in Nigeria. *Electronic Journal of Environmental, Agricultural and Food Chemistry*, *6*, 10.

Payne, W. J., & Carlson, A. B. (1957). Studies on Bacterial Utilization of Uronic Acids II. Growth Response and Oxidative Activity of Various Species. *Journal of Bacteriology*, *74*(4), 502–506. Retrieved from https://jb.asm.org/content/74/4/502.long

Rojas, R., Nishidomi, S., Nepomuceno, R., Oshiro, E., & de Cassia Café Ferreira, R. (2013). Glutamate transport and xanthan gum production in the plant pathogen *Xanthomonas axonopodis* pv. *citri*. *World Journal of Microbiology and Biotechnology*, *29*(11), 2173–2180. https://doi.org/10.1007/s11274-013-1383-4

Schatschneider, S., Persicke, M., Watt, S. A., Hublik, G., Pühler, A., Niehaus, K., & Vorhölter, F.-J. J. (2013). Establishment, in silico analysis, and experimental verification of a large-scale metabolic network of the xanthan producing *Xanthomonas campestris* pv. *campestris* strain B100. *Journal of Biotechnology*, *167*(2), 123–134. https://doi.org/10.1016/j.jbiotec.2013.01.023

Sitkin, B. V, Tsfasman, I. M., Stepnaya, O. A., & Kulaev, I. S. (2003). Intracellular Peptidoglycan Hydrolases of the Bacterium *Xanthomonas campestris* XL 1. *Biochemistry*, *68*(4).

Stoyanova, M., Vancheva, T., Moncheva, P., & Bogatzevska, N. (2014). Differentiation of *Xanthomonas* spp. causing bacterial spot in Bulgaria based on biolog system. *International Journal of Microbiology*, *2014*. https://doi.org/10.1155/2014/495476

Swings, J. C., & Civerolo, E. L. (Eds.). (1993). *Xanthomonas* (1st ed.). https://doi.org/10.1007/978-94-011-1526-1

Tang, D., He, Y., Feng, J., He, B., Jiang, B., Lu, G., & Chen, B. (2005). *Xanthomonas campestris* pv . *campestris* Possesses a Single Gluconeogenic Pathway that is Required for Virulence. *Journal of Bacteriology*, *187*(17), 6231–6237. https://doi.org/10.1128/JB.187.17.6231

Voloudakis, A. E., Reignier, T. M., & Cooksey, D. A. (2005). Regulation of resistance to copper in *Xanthomonas axonopodis* pv. *vesicatoria*. *Applied and Environmental Microbiology*, *71*(2), 782–789. https://doi.org/10.1128/AEM.71.2.782-789.2005

Vorhölter, F. J., Schneiker, S., Goesmann, A., Krause, L., Bekel, T., Kaiser, O., … Pühler, A. (2008). The genome of *Xanthomonas campestris* pv. *campestris* B100 and its use for the reconstruction of metabolic pathways involved in xanthan biosynthesis. *Journal of Biotechnology*, *134*(1–2), 33–45. https://doi.org/10.1016/j.jbiotec.2007.12.013

Yuan, Z., Wang, L., Sun, S., Wu, Y., & Qian, W. (2013). Genetic and Proteomic Analyses of a *Xanthomonas campestris* pv. *campestris* purC Mutant Deficient in Purine Biosynthesis and Virulence. *Journal of Genetics and Genomics*, *40*(9), 473–487. https://doi.org/10.1016/j.jgg.2013.05.003

Zhang, Z., & Chen, H. (2010). Fermentation performance and structure characteristics of xanthan produced by *Xanthomonas campestris* with a glucose/xylose mixture. *Applied Biochemistry and Biotechnology*, *160*(6), 1653–1663. https://doi.org/10.1007/s12010-009-8668-y

Zimaro, T., Thomas, L. L. L. L., Marondedze, C., Garavaglia, B. S. B. B. S., Gehring, C., Ottado, J., … Conesa, A. (2013). Insights into *Xanthomonas axonopodis* pv. *citri* biofilm through proteomics. *BMC Microbiology*, *13*(1), 186. https://doi.org/10.1186/1471-2180-13-186

Zimaro, T., Thomas, L., Marondedze, C., Garavaglia, B. S., Gehring, C., Ottado, J., & Gottig, N. (2011). The *Xanthomonas campestris* pv. *vesicatoria* citH gene is expressed early in the infection process of tomato and is positively regulated by the TctDE two-component regulatory system. *Molecular Plant Pathology*, *12*(1), 57–71. https://doi.org/10.1111/j.1364-3703.2010.00652.x
