# Supplementary material for: Genome-Scale Metabolic Model of Xanthomonas phaseoli pv. manihotis: An Approach to Elucidate Pathogenicity at the Metabolic Level
Source: Front Genet. 2020 Aug 11;11:837. doi: 10.3389/fgene.2020.00837 (PMC7432306; doi:10.3389/fgene.2020.00837)
Supplement: Supplementary file 2 [file Data_Sheet_2.docx]

**Supplementary Data**

- All related code, models and maps can be downloaded from GitHub: <https://github.com/davidoctaviobotero/Xpm_metabolic_model>
- Metabolic map of the central metabolism and carbohydrate sources of *Xpm* constructed in Escher (Tarazona et al., 2015): <https://github.com/davidoctaviobotero/Xpm_metabolic_model/blob/master/Maps/Xpm_Central_metabolism_map.json>
- Metabolic map of amino acid biosynthesis of *Xpm* constructed in Escher (King et al., 2015): <https://github.com/davidoctaviobotero/Xpm_metabolic_model/blob/master/Maps/Xpm-Aminoacid_map.json>
- Metabolic map of xanthan biosynthesis of *Xpm* constructed in Escher (King et al., 2015): <https://github.com/davidoctaviobotero/Xpm_metabolic_model/blob/master/Maps/Xpm-Xanthan_biosynthesis_map.json>
- Full metabolic model of *Xpm:* <https://github.com/davidoctaviobotero/Xpm_metabolic_model/blob/master/Models/Xam_Biomass%26Xanthan(completed_mar15)_model.json>
- Context Specific Metabolic Model of *Xpm* CIO151 EV: <https://github.com/davidoctaviobotero/Xpm_metabolic_model/blob/master/Models/Xam_void_CSM.mat>
- **Context Specific Metabolic Model of *Xpm* CIO151 *ΔrpfCGH*-EV:** [**https://github.com/davidoctaviobotero/Xpm_metabolic_model/blob/master/Models/Xam_rpfCGH_CSM.mat**](https://github.com/davidoctaviobotero/Xpm_metabolic_model/blob/master/Models/Xam_rpfCGH_CSM.mat)

**Supplementary Figures**

| 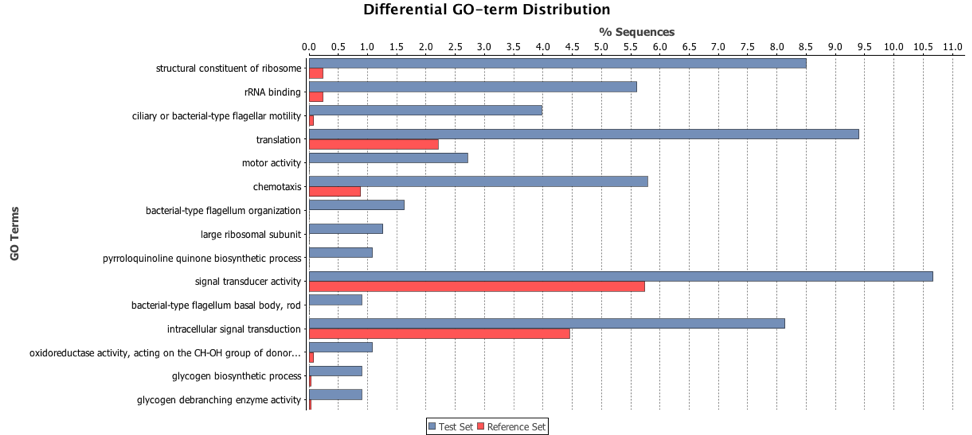 |
| --- |
| **Figure S1.** Over-represented gene ontologies upregulated by *rpfCGH* in *Xpm* using a Fisher Enrichment Analysis [Blast2GO]. Motor, flagellar and chemotaxis activities were among the most abundant, followed by signaling transduction processes. References set (red): proportion of GO-term in *Xpm* genome; Test set (blue): proportion of GO-term in the DEGs group of *Xpm*. |

| 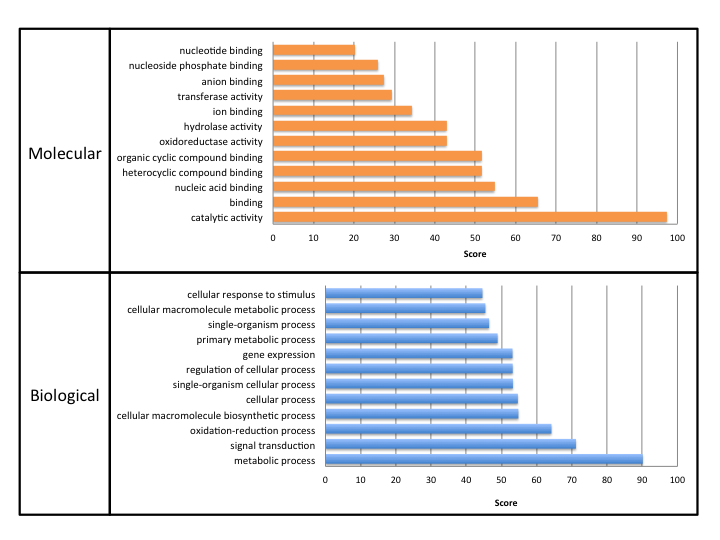 |
| --- |
| **Figure S2.** Overrepresented molecular functions and biological processes regulated by *rpfCGH* according to DEGs identified with NOISeqBIO. Scores were calculated by adding the highest hit similarity weighted by its EC number with the number of total GOs unified at the nearest node, to include the possibility of selecting parent nodes when several child nodes existed. |

| 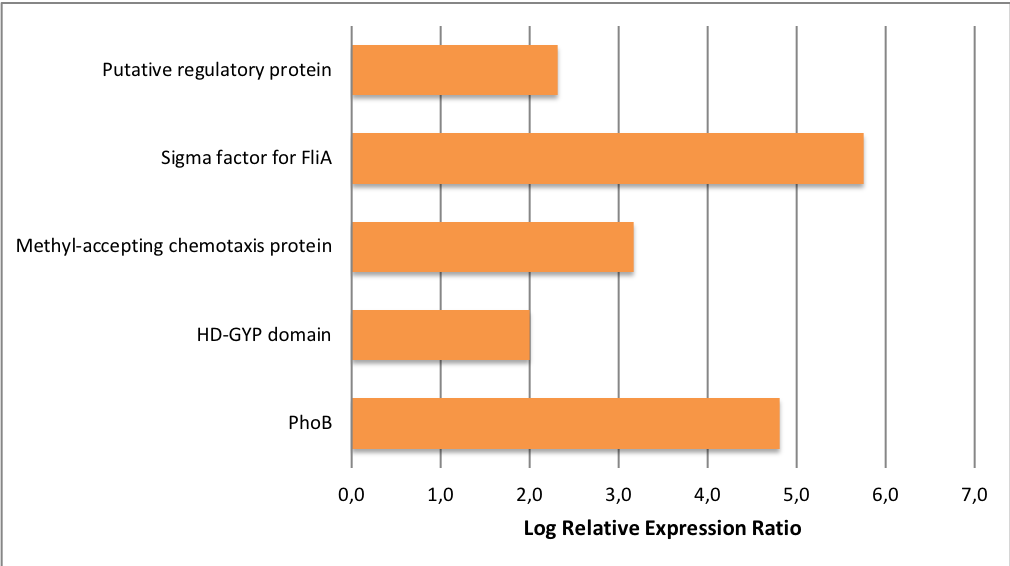 |
| --- |
| **Figure S3.** Relative expression ratio of upregulated genes validated through qRT-PCR using *gyrB* as the endogenous control. Bars indicate log relative expression ratios for genes that showed upregulation by *rpfCGH* in RNA-seq experiments and were evaluated by qRT-PCR. |

| 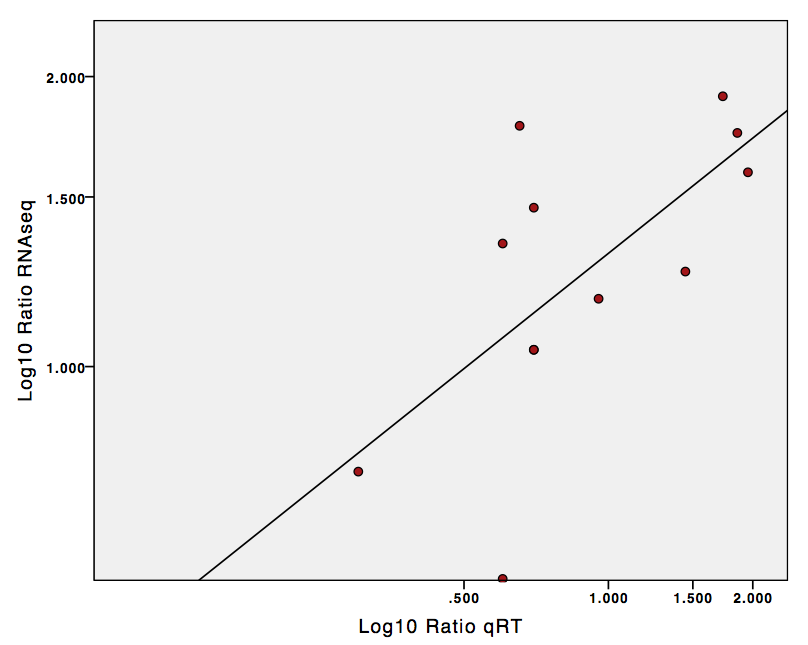 |
| --- |
| **Figure S4.** Correlation between RNA-Seq and qRT-PCR data for the validated DEGs. Pearson Coefficient showed a significant correlation between both datasets (α = 0.05). |

| 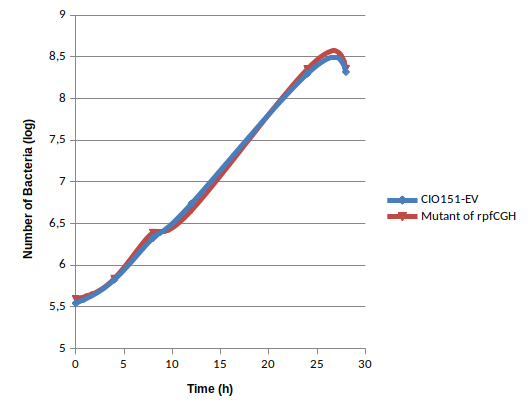 |
| --- |
| **Figure S5.**  Growth curve of *Xpm* in Phi ɸ liquid media. Bacteria strains were grown at 28ºC in a Phi ɸ liquid media for 28 hours at 28ºC with shaking at 200 rpm. Figure shows cell populations determined by measuring their colony forming units (CFU) over a period of time. |

| 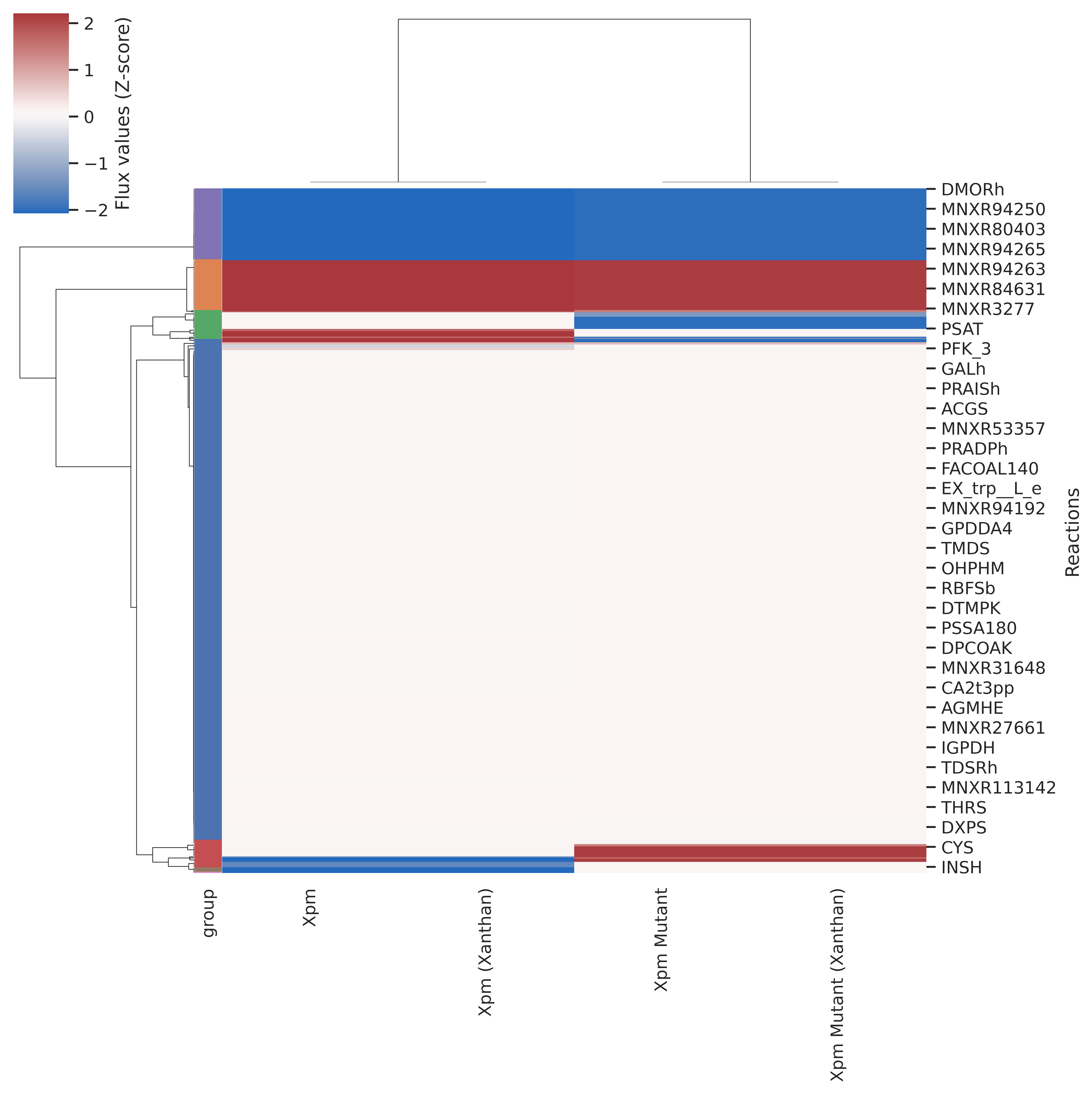 |
| --- |
| **Figure S6. Hierarchical Clustering Heatmap of *Xpm* strains differentiated in the QS system.** Comparisons between the flux values obtained by FBA of the two strains of *Xpm* (*Xpm* CIO151 EV and *Xpm* CIO151 *ΔrpfCGH*-EV)*,* using two different objective functions are shown. First, Z-scores were computed to normalize flux values. Then, the linkage matrix with Euclidean distance based on Ward method was used to construct the map. The dendrogram was colored for grouping purposes using a color threshold of 0.25. Finally, cluster maps of the two strains were calculated. The vertical axis represents the reactions and the horizontal axis the strains and conditions. Flux values for each reaction are colored according to its activity from white to green colors, positive values (white) correspond to forward direction of the reaction and negative values (dark green) correspond to reverse direction. **Abbreviations**. ***Xpm***: CSM of *Xpm* CIO151 EV using biomass as objective function; ***Xpm (Xanthan)***: CSM of *Xpm* CIO151 EV using biomass and xanthan as objective function; ***Xpm Mutant***: CSM of *Xpm* CIO151 *ΔrpfCGH*-EV using biomass as objective function; ***Xpm Mutant(Xanthan)***: CSM of *Xpm* CIO151 *ΔrpfCGH*-EV using biomass plus xanthan as objective function. |

| \| **Gene Product** \| **Gene ID** \| **Relative Expression Ratio** \| \| **Reported Functions in gram negative bacteria** \| \| --- \| --- \| --- \| --- \| --- \| \| RNA-Seq \| qRT-PCR \| \| Outer membrane efflux protein \| fig\|1185660.4.peg.405 \| 56 \| 72 \| Resistance against external toxins (antibiotics, host hormones and phytoalexins), copper resistance, and manganese homeostasis (Burse, Weingart, & Ullrich, 2004; C. Li, Tao, Mao, & He, 2011; Nishino, Latifi, & Groisman, 2006; Piddock, 2006; Ryan et al., 2007; Zhao, Li, Srikumar, & Poole, 1998). \| \| Hypothetical protein \| fig\|1185660.4.peg.2568 \| 60 \| 4.5 \| Unknown function \| \| M13-type metallopeptidase \| fig\|1185660.4.peg.2655 \| 39 \| 90 \| Cleaving of extracellular peptides: activation of a self-secreted peptide, disruption of a host-secreted peptide, aminoacid uptake (Kiely, O’Callaghan, Abbas, & O’Gara, 2008; Oetjen, Fives-Taylor, & Froeliger, 2001; Yanagisawa et al., 1988) \| \| ABC transporter \| fig\|1185660.4.peg.3022 \| 11 \| 5 \| Sharing similarity to RaxB involved in proteolysis associated with the T1SS (Da Silva et al., 2004) \| \| LysR-type transcriptional regulator \| fig\|1185660.4.peg.909 \| 22 \| 4 \| Phenolic compound catabolism, biofilm and *N*-acyl homoserine lactone production, resistance against antibiotics, ROS and low pH environments (Bernier, Nguyen, & Sokol, 2008; Parke, 1996; Qian et al., 2013; Srinivasan, Mondal, Venkataramaiah, Chauhan, & Rajamohan, 2013) \| \| TetR-type transcriptional regulator \| fig\|1185660.4.peg.1195 \| 29 \| 5 \| Antibiotic and osmotic stress resistance, regulation of quorum sensing, efflux pumps, and biofilm formation (Aramaki, Yagi, & Suzuki, 1995; Jeng et al., 2008; Pompeani et al., 2008; Ramos et al., 2005) \| \| Hsp90 \| fig\|1185660.4.peg.2960 \| 6 \| 2 \| Assembly, folding, delivery, and degradation of proteins during stressful conditions (Narberhaus 2002, Nakamoto and Vigh 2007) \| \| PhoB two-comp. system regulatory protein \| fig\|1185660.4.peg.880 \| 18 \| 28 \| Senses inorganic phosphate to regulate T3SS & T6SS in *E. tarda* (King et al., 2015) and virulence genes in *V. cholerae* (King et al., 2015). \| \| Putative signal protein with HD-GYP domain \| fig\|1185660.4.peg.531 \| 4 \| 4 \| Possible indirect regulation of genes through second messengers; virulence; stress response (Pratt, Ismail, & Camilli, 2010). \| \| Methyl-accepting chemotaxis protein \| fig\|1185660.4.peg.3088 \| 15 \| 9 \| Senses chemical attractants; colonization (Chakraborty, Sivaraman, Leung, & Mok, 2011). \| \| RNA pol. sigma factor for flagellar operon FliA \| fig\|1185660.4.peg.4580 \| 81 \| 54 \| Flagellar biosynthesis, virulence, biofilms, adherence, pectate lyases (Fuchs et al., 2010; Wilson et al., 2007). \| \| Putative regulatory protein of a two-comp. system \| fig\|1185660.4.peg.3529 \| 11 \| 5 \| Chemotaxis, adhesion, invasion, flagellar rotation (Z. Li et al., 2014). \| \| **Table S11.** Upregulated genes validated through qRT-PCR. The relative expression ratio was calculated using *gyrB* as an endogenous control in both biological replicates; the number indicates how many times more the unknown gene is being expressed with respect to the endogenous control. Significance values were calculated using a permutations test. \| \| \| \| \| |
| --- | --- | --- | --- | --- | --- | --- | --- | --- | --- | --- | --- | --- | --- | --- | --- | --- | --- | --- | --- | --- | --- | --- | --- | --- | --- | --- | --- | --- | --- | --- | --- | --- | --- | --- | --- | --- | --- | --- | --- | --- | --- | --- | --- | --- | --- | --- | --- | --- | --- | --- | --- | --- | --- | --- | --- | --- | --- | --- | --- | --- | --- | --- | --- | --- | --- | --- | --- | --- | --- | --- | --- | --- |
